# Complexation-induced resolution enhancement of 3D-printed hydrogel constructs

**DOI:** 10.1038/s41467-020-14997-4

**Published:** 2020-03-09

**Authors:** Jiaxing Gong, Carl C. L. Schuurmans, Anne Metje van Genderen, Xia Cao, Wanlu Li, Feng Cheng, Jacqueline Jialu He, Arturo López, Valentin Huerta, Jennifer Manríquez, Ruiquan Li, Hongbin Li, Clément Delavaux, Shikha Sebastian, Pamela E. Capendale, Huiming Wang, Jingwei Xie, Mengfei Yu, Rosalinde Masereeuw, Tina Vermonden, Yu Shrike Zhang

**Affiliations:** 1 Division of Engineering in Medicine, Department of Medicine, Brigham and Women’s Hospital, Harvard Medical School, 65 Landsdowne Street, Cambridge, MA 02139 USA; 2 The Affiliated Stomatology Hospital, Zhejiang University School of Medicine, 310003 Hangzhou, P. R. China; 3 Key Laboratory of Oral Biomedical Research of Zhejiang Province, 310003 Hangzhou, P. R. China; 4 Department of Pharmaceutics, Utrecht Institute for Pharmaceutical Sciences (UIPS), Science for Life, Utrecht University, Universiteitsweg 99, 3508 TB Utrecht, The Netherlands; 5 Department of Pharmacology, Utrecht Institute for Pharmaceutical Sciences (UIPS), Utrecht University, Universiteitsweg 99, 3508 TB Utrecht, The Netherlands; 6 Department of Surgery-Transplant and Holland Regenerative Medicine Program, University of Nebraska Medical Center, 985965 Nebraska Medical Center, Omaha, NE 68198 USA

**Keywords:** Soft materials, Bioinspired materials, Polymers

## Abstract

Three-dimensional (3D) hydrogel printing enables production of volumetric architectures containing desired structures using programmed automation processes. Our study reports a unique method of resolution enhancement purely relying on post-printing treatment of hydrogel constructs. By immersing a 3D-printed patterned hydrogel consisting of a hydrophilic polyionic polymer network in a solution of polyions of the opposite net charge, shrinking can rapidly occur resulting in various degrees of reduced dimensions comparing to the original pattern. This phenomenon, caused by complex coacervation and water expulsion, enables us to reduce linear dimensions of printed constructs while maintaining cytocompatible conditions in a cell type-dependent manner. We anticipate our shrinking printing technology to find widespread applications in promoting the current 3D printing capacities for generating higher-resolution hydrogel-based structures without necessarily having to involve complex hardware upgrades or other printing parameter alterations.

## Abstract (web summary)

Three-dimensional (3D) hydrogel printing enables production of volumetric architectures but achieving good resolutions for miniaturized features remains challenging. Here the authors demonstrate shrinking of a printed structure by immersing a 3D-printed patterned hydrogel consisting of a hydrophilic polyionic polymer network in a solution of polyions of the opposite net charge.

## Introduction

Three-dimensional (3D) printing has become a collection of enabling biofabrication technologies to generate volumetric structures featuring high complexity through robotically controlled dispensing or stabilization processes^1,2^. The programmed fabrication further makes the procedures highly reproducible. As such, 3D printing is finding widespread applications in biomedicine including, but not limited to, tissue engineering, microphysiological systems, and biomedical devices^3^. Nevertheless, existing printing strategies all have their minimally producible resolutions, which are factors of multiplexed parameters, such as the printer hardware and ink properties^4^. For example, in extrusion printing using hydrogels as inks, the resolutions are typically sub-millimeter for the dispensed microfibers^4^. The same holds true for microfluidic coaxial printing, where the diameters of the created hollow microfibers usually fall in the range of a couple hundred micrometers or larger^4,5^. Although some other printing strategies, such as those based on light (e.g., two-photon lithography) can achieve varying degrees of higher resolutions^6^, their instrumentation is usually complicated limiting the broader adoption for general use.

To this end, efforts have conventionally been focused on improving the printer hardware or ink properties. Examples in extrusion printing include using smaller nozzles or using inks of high viscosity values, both of which inevitably elevate the need for much higher forces during the dispensing processes, inducing significantly increased shear stresses on the encapsulated cells. Even though, the resolution improvements are still limited. Meanwhile, the progress in printer hardware or tuning the ink formulations may not always be straightforward, leaving these methods still impractical for some applications.

More recently, an implosion fabrication method was proposed^7^, in which a swollen hydrogel matrix was used to photopattern metals, semiconductors, and biomolecules, followed by acid-driven shrinking to achieve nano-sized structures. While efficient for two-photon lithography, this method is not amenable to most other 3D printing modalities due to the necessity of a pre-existing, swollen hydrogel matrix to allow anchoring points for secondary biomolecules or inorganic species subsequently patterned in the volumetric space. Further, such shrinking is unstable and would revert once the stimulus is removed. Finally, the harsh implosion conditions (i.e., low pH) utilized are largely incompatible with living cell-based applications.

Here, we propose a strategy of complexation-induced resolution enhancement in 3D printing that we term as shrinking printing, through post-treatment of the printed structures, for resolution improvement without requiring changing any of the printer hardware or much of the ink compositions. In particular, we select inks that are anionic, such as those based on commonly used hyaluronic acid methacrylate (HAMA)^8^, gelatin methacryloyl (GelMA)^9^, and alginate^10^. Following standard printing procedures, we subject the HAMA-, GelMA-, or alginate-based hydrogel constructs to immersion in a polycationic chitosan solution. Through charge complexation and subsequent expulsion of water from the gels, these printed constructs are found to reduce in their linear dimensions in various degrees. We conduct our proof-of-concept studies with several 3D printing techniques, including direct extrusion printing, sacrificial printing, and microfluidic hollow fiber printing, where successful shrinking is observed in all cases. Additionally, we prove the wide applicability of our method by demonstrating shrinking of printed polycationic chitosan-based hydrogel constructs, with polyanionic alginate. We finally demonstrate that selected types of cells embedded in the printed hydrogel matrices remain viable upon successive shrinking in contrast to a single, longer shrinking procedure, further revealing the potential applications of our shrinking printing method in the presence of living cells. We anticipate wide adoption of this technology in future printing of acellular or cellularized structures with further optimizations.

## Results

### Shrinking behaviors of polyionic hydrogels

**Fig. 1:**
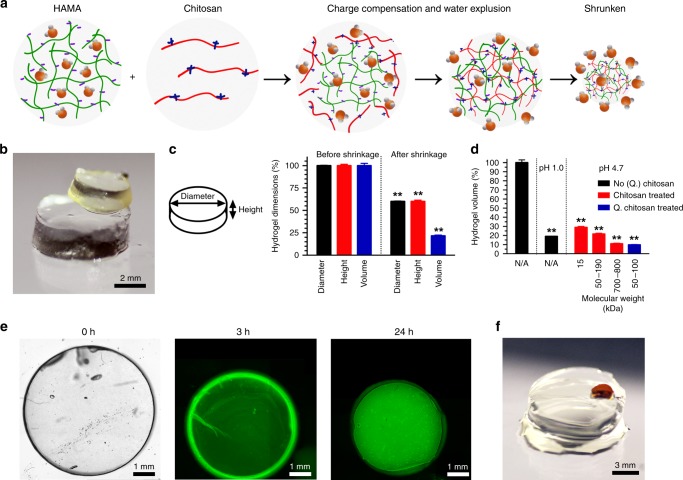
The general mechanism of complexation-induced hydrogel shrinking. **a** Schematics showing the shrinking effect based on charge compensation. **b** Photograph showing size change of fabricated HAMA hydrogel (1.0 w/v%) before (lower) and after (upper) shrinking in 2.0 w/v% MM_w_ chitosan dissolved in 1.0 v/v% acetic acid aqueous solution. **c** Schematic representation of a HAMA hydrogel disc together with the dimensions and corresponding quantitative analyses showing the dimensions and volume changes before and after shrinking in 2.0 w/v% MM_w_ chitosan dissolved in 1.0 v/v% acetic acid aqueous solution. **d** Corresponding quantitative analyses showing the shrinking of HAMA hydrogels in perchloric acid solution (pH = 1.0) or in 1.0 v/v% acetic acid aqueous solutions (pH = 4.7) with 2.0 w/v% of chitosan of different molecular weights and types. **e** Confocal images showing the diffusion of FITC-Q. chitosan solution in PBS into a 2.0 w/v% HAMA hydrogel at 3 and 24 h of shrinking. The bright-field image at 0 h serves as the size reference of the initial hydrogel. **f** Photograph showing size change of HM_w_ chitosan hydrogels (2.0 w/v%), where the lower one was swollen in 1.0 v/v% acetic acid aqueous solution and the upper one was shrunken in 2.0 w/v% alginate in 1.0 v/v% acetic acid aqueous solution. **P < 0.01; one-way ANOVA (**c**, **d**, compared with the values of corresponding as-prepared samples); mean ± s.d, (*n* = 3).

The carboxyl groups present in HAMA render the hydrogel negatively charged under physiological conditions. When a HAMA hydrogel is placed in the presence of polycations of high charge densities, such as chitosan, a type of glucosamine featuring reasonable biocompatibility and densely cationic nature due to the abundantly available amino groups^11^, charge compensation occurs leading to expulsion of water and eventual size reduction of the HAMA hydrogel (Fig. 1a and Supplementary Fig. 1). Indeed, this phenomenon was previously observed for microgels^12^. We also recently demonstrated, that when a solution of positively charged lysozyme was introduced to microfluidics-fabricated HAMA microspheres, the size of these microspheres was decreased due to a mechanism akin to complex coacervation, facilitating their use in drug delivery^13^.

To prove the concept, we first fabricated cylindrical HAMA hydrogels (1.0 w/v%, *D* = 6 mm, *H* = 2 mm, ~56.5 µL in volume). When immersed in 2.0 w/v% chitosan (*M*_w_: 50–190 kDa) dissolved in 1.0 v/v% acetic acid aqueous solution for 24 h, the hydrogels shrank ~60% in height and diameter, leading to a reduction to 21% in volume as compared to the original constructs (Fig. 1b and Fig. 1c). The changes of concentrations of HAMA hydrogels before and after complete shrinkage in chitosan (*M*_w_: 700–800 kDa) were subsequently evaluated. Our calculations revealed that the concentrations increased from the initial 0.5–2.5 w/v% to 7.9–12.1 w/v% after shrinking (Supplementary Fig. 2). Scanning electron microscopy (SEM) images of the HAMA structures before and after shrinking in chitosan (*M*_w_: 700–800 kDa) are illustrated in Supplementary Fig. 3. Although the concentrations of HAMA in the shrunken hydrogels were much higher than those of the initial constructs (Supplementary Fig. 2), water was still the main constituent maintaining their hydrogel nature for various relevant applications.

Considering that the molecular weight of chitosan might affect the shrinkage extent, we compared chitosan of different molecular weights (low-molecular weight, LM_w_: 15 kDa; medium-molecular weight, MM_w_: 50–190 kDa; high-molecular weight, HM_w_: 700–800 kDa). All chitosan types had a similar degree of deacetylation (85%). As shown in Fig. 1d, the HAMA hydrogels (1.0 w/v%) at pH = 1.0 shrank to 19.0 ± 0.2% of their original size, while the ones in 2.0 w/v% LM_w_ chitosan, MM_w_ chitosan, and HM_w_ chitosan in 1.0 v/v% acetic acid aqueous solutions (pH = 4.7) shrank to 28.9 ± 1.1, 21.6 ± 0.9, and 11.0 ± 0.7%, respectively. In addition to the molecular weight, the charge density of the shrinking agent might be an important factor affecting the shrinkage degree. Therefore, we compared three types of chitosan with different degrees of deacetylation (72.5%, 77.8%, and 94.6%) but similar molecular weights (50–250 kDa). As shown in Supplementary Fig. 4, the HAMA hydrogels (1.0 w/v%) in 1.0 v/v% acetic acid aqueous solution (pH = 4.7) shrank to 55.1 ± 1.5% of their original size, and the ones in 2.0 w/v% chitosan in 1.0 v/v% acetic acid aqueous solutions shrank to 23.5 ± 1.0, 18.5 ± 0.8, and 12.6 ± 0.7% for chitosan with different degrees of deacetylation (72.5%, 77.8%, and 94.6%), respectively. Moreover, the shrinkage ratio with quaternary ammonium salt chitosan (Q. chitosan, *M*_w_: 50–100 kDa, 90% of deacetylation degree, 95% of quaternization degree) was observed to be the highest among all chitosan types, at 9.7 ± 0.4% (Fig. 1d).

These results indicated that both the average polymer chain length and the average charge per monomer of the chosen cationic polymer, i.e., chitosan, influenced the extent of the HAMA hydrogels. The strength of electrostatic complexation between two oppositely charged polymers is known to be dependent on the chain length and charge density (i.e., amount of monomers/charges in a single polymer chain)^14,15^. These observations coupled to the fact that shrinking with polycations resulted in lower-volume (i.e., more heavily dehydrated) hydrogels than when compared to a similar hydrogel incubated in a buffered pH 1.0 solution bring us to the conclusion that, the hydrogel shrinks due to the formation of a complex coacervate-like structure between the oppositely charged hydrophilic polymer network and the free polymer. Dehydration is commonly seen in both complex coacervation and precipitation in an electrostatic strength-dependent manner^14,15^. The characteristics of all types of chitosan used in this set of experiments and the shrinkage ratios of HAMA hydrogels (1.0 w/v%) are listed in Supplementary Table 1. When the deacetylation degree and thus the average positive charge per monomer remained constant (i.e., 85% of deacetylation degree, +1 per 271.8 Da of charge density), the higher molecular weight of chitosan was, the higher degree of shrinking was observed. The average charge density of every chitosan monomer would also be of great importance; the higher the charge density (+1 per 328.8 Da, +1 per 298.7 Da, +1 per 238.3 Da, for different degrees of deacetylation [72.5%, 77.8%, 94.6%] of chitosan, respectively), the higher the shrinkage ratio.

Moreover, we made two types of bulk HAMA hydrogels (2.0 w/v%) of similar aspect ratios to study effects of the initial hydrogel volume on hydrogel shrinking (cylinders, *D* = 6 mm, *H* = 2 mm, ~56.5 µL, or *D* = 8 mm, *H* = 3.4 mm, ~170.9 µL). As shown in Supplementary Fig. 5a, b, the thicker structures took longer to shrink, but shrank to a similar degree as the thinner structures did (Supplementary Fig. 5c). To investigate the shrinking process, we visualized the polycation diffusion using fluorescein isothiocyanate-labeled Q. chitosan (FITC-Q. chitosan), by incubating 2.0 w/v% HAMA cylinders in a 2.0 w/v% FITC-Q. chitosan solution, and after 3 and 24 h, observations were made with confocal microscopy (Fig. 1e). The results revealed that FITC-Q. chitosan diffused into the entire structure and complexed with the polyanion (HAMA) already at 3 h, yet with most of the FITC-Q. chitosan still located at the periphery. However, after complete shrinking at 24 h, we observed a homogeneous distribution of FITC-Q. chitosan throughout the cylinder, indicating that the shrinking agent fully penetrated the hydrogel matrix, and that the relatively low weight percentage of HAMA used for forming the hydrogel did not hamper the diffusion of large polymer chains of chitosan into the matrix. Subsequent release of FITC-Q. chitosan from the fully shrunken hydrogels was quantified in phosphate-buffered saline (PBS) and followed for 21 days (Supplementary Fig. 6a, b). Only in the first few days, release of FITC-Q. chitosan was observed, which can be attributed to the loss of FITC-Q. chitosan loosely bound (non-ionically) to the surfaces of the hydrogels that was washed off rapidly during the incubation. After the first few days, no further release was observed. The hydrogel volumes did not change over time, indicating that the shrunken constructs remained stable without further significant release of chitosan (Supplementary Fig. 6c).

Since alginate is one of the most commonly used hydrogels in 3D printing, we investigated whether our shrinking process was also compatible with alginate hydrogels (2.0 w/v%). After physical crosslinking in a CaCl_2_ solution (0.3 M), the alginate cylinders (*D* = 6 mm, *H* = 2 mm, ~56.5 µL) were obtained and washed in de-ionized water, or shrunken in 2.0 w/v% of chitosan of different molecular weights and types, as shown in Supplementary Fig. 7. The alginate hydrogels in water swelled to 105.9 ± 2.5%, and when incubated in 1.0 v/v% acetic acid aqueous solution, the cylinders shrank to 51.2 ± 1.4% of their original volume, whereas the ones in LM_w_ chitosan, MM_w_ chitosan, and HM_w_ chitosan solutions shrank to 37.6 ± 1.2%, 28.9 ± 1.1%, and 27.7 ± 1.0%, respectively. Moreover, the shrinkage degree in Q. chitosan was validated at 25.4 ± 3.9%, similar to that shrunken by the HM_w_ chitosan. These results indicated that alginate, as a negatively charged polymer similar to HAMA, could also shrink in a polycationic solution using our complexation-based technique.

To investigate whether this approach was generally applicable to polycationic inks as well, we prepared chitosan-based cylindrical hydrogels for further study. HM_w_ chitosan hydrogel (2.0 w/v%) cylinders (*D* = 6 mm, *H* = 2 mm, ~56.5 µL) crosslinked with different concentrations of glutaraldehyde were incubated either in 1.0 v/v% acetic acid aqueous solution or 2.0 w/v% alginate in 1.0 v/v% acetic acid aqueous solution. As shown in Supplementary Fig. 8, the chitosan hydrogels swelled in 1.0 v/v% acetic acid aqueous solution and shrank when incubated in the presence of alginate, validating that this approach is applicable to hydrogels formed by polycationic polymers as well. Figure 1f visually shows the swollen chitosan hydrogel (2.0 w/v%) in 1.0 v/v% acetic acid aqueous solution (lower construct) and the shrunken hydrogel (upper construct) in 2.0 w/v% alginate in 1.0 v/v% acetic acid aqueous solution when the molar ratio of glutaraldehyde to chitosan chains was 5:1. With increasing amounts of glutaraldehyde, lower swelling in acetic acid solution and lower shrinking in alginate solution were observed, which can be ascribed to the removal of the positive charges in chitosan by the reaction of the primary amines with glutaraldehyde.

In general, positively charged hydrogels are less frequently utilized and are typically adopted for situations where their charge is key for the application (a feature that would be negated by our complexation-based technique). Moreover, cationic hydrogels in general are more cytotoxic^16^ and therefore less commonly used for applications intended to grow cells in before charge neutralization. Therefore, we intended to focus more on the shrinking abilities of negatively charged hydrogels (i.e., HAMA, GelMA, and alginate), with a brief demonstration also on the shrinking of positively charged hydrogels (chitosan).

### Shrinking behaviors of extrusion-printed structures

**Fig. 2:**
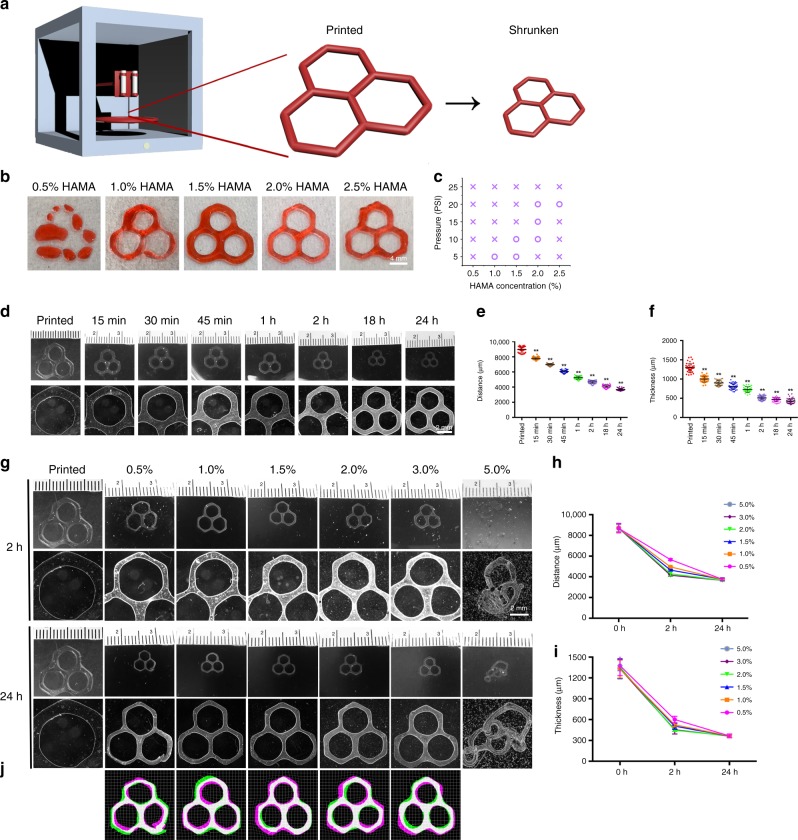
Direct extrusion printing of HAMA constructs and their shrinking behaviors. **a** Schematics showing the concept of shrinking printing, where a printed hydrogel structure is post-treated to reduce its size and achieve higher resolution. **b**, **c** Printability mapping of HAMA inks at different concentrations and extrusion pressures. **d** Photographs (upper) and micrographs (lower) showing size changes of printed HAMA hexagons (2.0 w/v%) immersed in 2.0 w/v% HM_w_ chitosan dissolved in 1.0 v/v% acetic acid aqueous solution during the 24h shrinking process. **e**, **f** Corresponding quantitative analyses of size changes of the printed HAMA hexagons (2.0 w/v%), include **e** side-to-side distance and **f** thickness, during the 24h shrinking process. **g** Photographs (upper) and micrographs (lower) showing size changes of printed HAMA hexagons (2.0 w/v%) at 2 h and 24 h of shrinking in different concentrations of HM_w_ chitosan (0.5–5.0 w/v%) dissolved in 1.0 v/v% acetic acid aqueous solution, **h**, **i** Corresponding quantitative analyses of size changes in **h** side-to-side distance and **i** thickness. **j** Vector-field maps comparing the 24-h shrinking images (magenta) to the corresponding 2-h shrinking images (green) by a B-spline-based non-rigid registration algorithm, where the overlaps appear in white and the grids show local distortions. Note that the 2-h shrinking images have been rescaled to match the sizes of the 24-h shrinking images to enable comparisons. **P < 0.01; one-way ANOVA (**e**, **f**, compar**e**d with the values of corresponding as-prepared samples); mean ± s.d. (**e**, **f**, *n* = 40; **h**, **i**, *n* = 10).

Here, we sought to demonstrate that the same process might be adapted to the printed hydrogel structures to achieve resolution improvement otherwise only possible through changes in printing parameters (such as nozzle size, pressure, and/or nozzle moving speed). We first evaluated the printability of HAMA inks (0.5–2.5 w/v%) in extrusion printing by depositing hexagonal patterns (Fig. 2a). Our printability mapping (Fig. 2b, c) suggested an optimal HAMA ink formulation of 2.0 w/v%, which was chosen for the subsequent experiments using extrusion printing. Young’s moduli of the resulting HAMA hydrogels after photocrosslinking were evaluated (Supplementary Fig. 9a), where the values ranged from 1.7 ± 0.2 to 24.1 ± 3.3 kPa. These values are in accordance with those reported in literature for HAMA hydrogels^17^.

We immersed our printed structures in 2.0 w/v% HM_w_ chitosan dissolved in 1.0 v/v% acetic acid in deionized water (pH = 4.7). It was observed that the hexagonal HAMA hydrogels shrank to smaller sizes as a function of time (Supplementary Movie 1). As illustrated in Fig. 2d–f, the distance (side-to-side) of the hexagons was reduced from 893 ± 377 to 3674 ± 163 μm in 24 h, or 41.1% of the original size (Fig. 2e). The thickness of the fibers in the hexagons also decreased from 1295 ± 113 to 424 ± 68 μm, or 32.7% of the original size (Fig. 2f). The Young’s modulus of HAMA increased from 15.9 ± 1.8 kPa to 24.3 ± 3.0 kPa in the 2.0 w/v% HAMA group (Supplementary Fig. 9a). Shrinking of the HAMA hydrogels was also validated by measuring their volume changes, where the 2.0 w/v% HAMA hydrogel shrank to ~20% of the original volume after chitosan complexation for 24 h (Supplementary Fig. 9b).

We subsequently investigated how different concentrations of HM_w_ chitosan (0.5, 1.0, 1.5, 2.0, 3.0, or 5.0 w/v%) dissolved in 1.0 v/v% acetic acid affected the shrinking of the printed HAMA (2.0 w/v%) structures. As revealed in Fig. 2g–i and Supplementary Fig. 10, at 2 h of shrinkage, the side-to-side distances of the hexagons decreased to a range between 4144 ± 98 μm (3.0 w/v% of HM_w_ chitosan) and 5664 ± 91 μm (0.5 w/v% of HM_w_ chitosan), or 47.6–65.1% of the original size. After 24 h of immersion, the side-to-side distance of the hexagons was further reduced to ~3700 μm, while the thickness of the fibers shrank from the initial 1350 μm to 360 μm. Distinct from those at 2 h of shrinking, neither the distance of the hexagons nor the thickness of the fibers indicated significant differences among the concentrations of HM_w_ chitosan, suggesting that the higher concentration of chitosan led to faster shrinking but the final gel volume could be reached in 24 h or earlier. It should be noted though, that the printed structures in the 5.0 w/v% HM_w_ chitosan group became irregular likely due to the high viscosity of the chitosan solution at this concentration that prevented uniform shrinking of the patterns. Since the fidelity of shrinking is important for retaining the printed structures, here we visualized the distortions of the 2-h shrinking and 24-h shrinking images by superimposing the 2-h shrinking images on top of the corresponding 24-h shrinking images^18^. As shown in Fig. 2j, the distortions (indicated by background grids) were deemed to be within an acceptable range, indicating that only little deformation of the total structure had taken place during the shrinking.

Considering that chitosan was dissolved in 1.0 v/v% acetic acid at a pH value of 4.7, we subsequently evaluated the possibility of utilizing Q. chitosan, which easily dissolves in deionized water and aqueous solutions at physiological pH^19^, as also used for shrinking non-printed hydrogel constructs above. As a control, we initially dissolved Q. chitosan in 1.0 v/v% acetic acid aqueous solution and investigated the shrinking behavior of the printed 2.0 w/v% HAMA structures (Supplementary Figs. 11a, 12). The results were similar to those obtained with regular HM_w_ chitosan solutions. The shrinking rate was proportional to the concentration of Q. chitosan and reached equilibrium at or before 24 h. Remarkably, the hexagons in 5.0 w/v% Q. chitosan were able to retain their shape and shrank to 40.8 ± 1.8% in side-to-side distance and 24.1 ± 2.6% in thickness as compared to the original dimensions, since the viscosity of Q. chitosan solution was much lower than that of the chitosan solution at this concentration, which facilitated uniform shrinking. When we shifted to the use of Q. chitosan in de-ionized water, the rate of shrinking seemed to become slightly slower as compared to using Q. chitosan or HM_w_ chitosan in acetic acid aqueous solution, where the side-to-side distance was reduced to 62.0–99.3% and the thickness to 52.1–99.5% of the original constructs after 2 h of incubation (Supplementary Figs. 13a and 14). Nevertheless, when the time was extended to 24 h, hydrogel sizes shrank down to 41.2–56.4% for the side-to-side distances and 33.0–45.9% for the thicknesses, both similar to the degrees of shrinking with HM_w_ chitosan or Q. chitosan in 1.0 v/v% acetic acid aqueous solution. Moreover, the distortions of the 2-h shrinking and 24-h shrinking images were also visualized (Supplementary Figs. 11b and 13b).

To illustrate that our method truly works in all three spatial dimensions, we further printed a HAMA (2.0 w/v%) pyramid frame in a gelatin supporting matrix (1.5 w/v%) followed by UV crosslinking (Supplementary Fig. 15a)^8^. After photocrosslinking of HAMA, the gelatin was heated and stepwise replaced with water. Finally, the 3D-printed pyramidal frame structure was incubated in 1.0 w/v% HM_w_ chitosan in 1.0 v/v% acetic acid aqueous solution for 24 h to achieve complete shrinking. As shown in Supplementary Fig. 15b, c, the printed HAMA pyramid shrank uniformly in every direction (length, height, thickness), to about 60% in linear dimensions those of the original constructs.

To eliminate the effect caused by changes in pH, we further incubated the samples in aqueous solutions of HM_w_ chitosan and Q. chitosan (both dissolved at 2.0 w/v% in 1.0 v/v% acetic acid aqueous solution), or an aqueous solution of pH = 1.0 (adjusted using perchloric acid without chitosan). The volume of the HAMA hydrogel in perchloric acid was still significantly larger than those incubated in the chitosan solutions (Supplementary Fig. 16a), verifying that the mechanism of shrinking with chitosan was not purely related to neutralizing the negative charges on the HAMA molecules. The Young’s moduli post-shrinking as expected, showed an opposite trend with their volumes (Supplementary Fig. 16b). We next evaluated the shrinking behavior of the printed HAMA structures (2.0 w/v%) in acetic acid aqueous solutions of different pH values. During the experiments, we found that the sizes of the printed HAMA hexagons shrank in acetic acid solutions of different pH values, where the lower the pH, the smaller the size, which yet were still larger than those shrunken with 2.0 w/v% HM_w_ chitosan in 1.0 v/v% acetic acid aqueous solution (Supplementary Fig. 17). The pH-induced shrinking was reversible, initially causing structures incubated in acid to shrink but recovering to the original printed size when subsequently placed into de-ionized water (Supplementary Fig. 18). In contrast, the chitosan-driven shrinking was stable during the investigated time frame attributed to the complexation process, consistent with our stability analysis on the bulk HAMA constructs (Supplementary Fig. 6). Therefore, the neutralization of charges by changes in pH could lead to a certain degree of shrinkage, but charge compensation by polyelectrolyte complexation of the polyanionic polymers and the polycationic hydrogels resulted in irreversible shrinkage in an electrostatic binding strength-dependent manner (also see Fig. 1d).

Interestingly, we found that the printed HAMA structures could shrink in cell culture medium as well. When subjecting the HAMA hydrogels to Q. chitosan dissolved in Dulbecco’s modified Eagle medium (DMEM, 2.0 w/v%), the constructs shrank to approximately half of their thickness and about 75% in size-to-size distance (Supplementary Fig. 19), although the shrinking rate was slower compared to those in acid or in water. This observation could be attributed to the higher ionic strength of DMEM (~170 mM) as compared to the 0–20 mM found in de-ionized water, where ionic strength of the medium dampens electrostatic interactions between positive and negative moieties. Nevertheless, our results indicated that shrinking of the printed constructs in the presence of cells is possible.

Printed alginate hexagons (2.0 w/v%) were physically crosslinked by 0.3-M CaCl_2_ (Supplementary Fig. 20), after which they were shrunken in 1.0 v/v% acetic acid aqueous solution, or in 2.0 w/v% chitosan of different molecular weights and types in 1.0 v/v% acetic acid aqueous solution. The corresponding quantitative analyses of size changes of the printed alginate hexagons are shown in Supplementary Fig. 21a for side-to-side distances and in Supplementary Fig. 21b for thicknesses.

Finally, we printed chitosan hexagons (2.5 w/v%), which were subsequently crosslinked by glutaraldehyde (800 μM). As expected, the printed chitosan hexagons shrank in 2.0 w/v% alginate in 1.0 v/v% acetic acid aqueous solution (Supplementary Fig. 22a), where the thickness and side-to-side distance changes were further reflected upon in Supplementary Fig. 22b.

### Shrinking bahaviors of sacrificially printed microchannel-embedded constructs

**Fig. 3:**
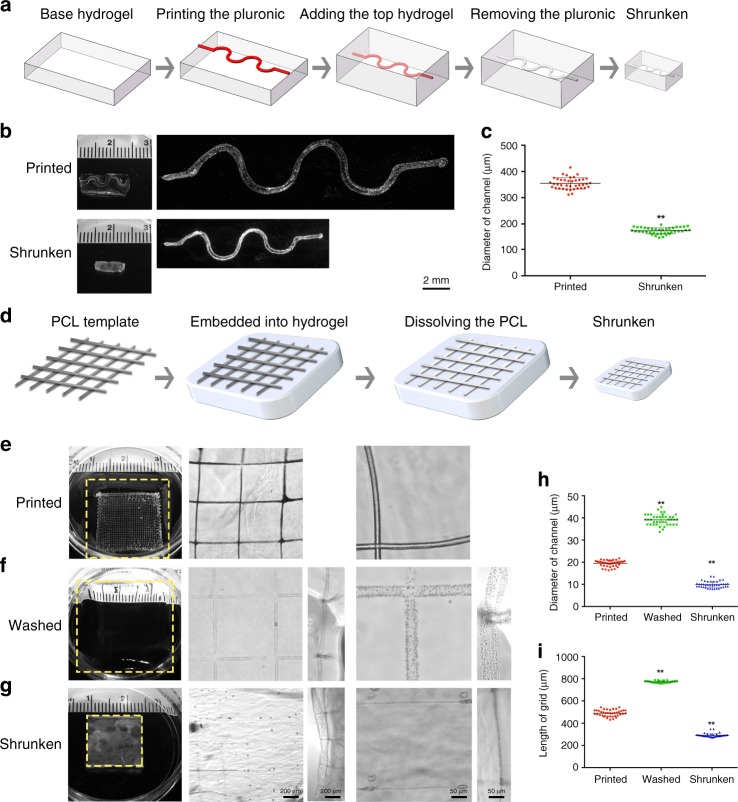
Sacrificial printing of microchannel-embedded HAMA constructs and their shrinking behaviors. **a** Schematic of the sacrificial printing process using an extrusion-printed Pluronic F-127 microfiber as the fugitive ink and subsequent shrinking of the construct. **b** Photographs showing the size change of the HAMA construct (2.0 w/v%) along with the embedded microchannel before (upper) and after (lower) 24 h of shrinkage in 2.0 w/v% HM_w_ chitosan dissolved in 1.0 v/v% acetic acid aqueous solution. **c** Corresponding quantitative analysis of diameter change of the microchannel before and after shrinking. **d** Schematic of the sacrificial printing process using a MEW-printed PCL grid as the fugitive ink and subsequent shrinking of the construct. **e**–**g** Photographs (left) and micrographs (right) showing the size change of the HAMA construct (2.0 w/v%) along with the embedded microchannel printed (**e**), washed (**f**) and shrunken (**g**) in 2.0 w/v% HM_w_ chitosan dissolved in 1.0 v/v% acetic acid aqueous solution (24 h). **h**, **i**, Corresponding quantitative analyses of changes in **h** diameter of the microchannel and (**i**) length of the grid before and after shrinking. **P < 0.01; two-tailed Student’s *t*-test (**c**), one-way ANOVA (**h**, **i**, compared with the corresponding as-printed structures); mean ± sd (*n* = 40).

Once we validated the hypothesis that electrostatic complexation could enable efficient size reduction of extrusion-printed HAMA patterns, we explored if the same concept can be applied to other commonly used 3D printing strategies. Again, we aimed to achieve improved resolution without needing to adjust the printing hardware or parameters. Among all approaches, sacrificial printing is a frequently used method to generate hollow, perfusable microchannels within hydrogel constructs. These microchannels would serve as biomimetic cannular structures to emulate human tissues, such as the vasculature^20^ or the proximal tubules found in kidneys^21^. A Pluronic F127 solution is thermosensitive, meaning that it flows at low temperatures but forms a solid hydrogel at elevated temperatures. As such, it has been frequently used as a fugitive ink, serving as a template during printing that can later be selectively removed from the hydrogel matrices to create hollow microchannels^21,22^ (Fig. 3a). We produced HAMA hydrogel constructs with a channel (2.0 w/v%) using Pluronic-based sacrificial printing (Fig. 3b). Measuring the diameter of the microchannels after removing the Pluronic fugitive template showed a value of 355 ± 21 μm, in accordance with those previously fabricated under similar conditions^21,22^. When the entire constructs were immersed in 2.0 w/v% HM_w_ chitosan in 1.0 v/v% acetic acid aqueous solution for 24 h, a gel size reduction was observed with associated decrease in the microchannel diameter to 174 ± 12 μm, or 49.0 ± 3.4% of the original size (Fig. 3b, c). The distortions between pre-shrinking and post-shrinking patterns were visualized and showed negligible changes in original shape (Supplementary Fig. 23).

While sacrificial printing using Pluronic as the fugitive ink renders the fabrication of hydrogel-embedded microchannels convenient, the channel sizes are typically limited to a few hundred micrometers or larger. Although with our unique shrinking strategy we could further reduce the channel diameter by a factor of 2, creating microchannels in the sub-100 μm scale with conventional sacrificial printing, relying on fugitive hydrogel inks is still challenging. More recently, melt electrospinning writing (MEW) has attracted increasing attention due to its ability to deposit well-defined meshes with filament sizes ranging from few micrometers down to sub-micron levels^23,24^. MEW-enabled sacrificial printing to generate microvascular networks has also been reported^25^. To further demonstrate the utility of our shrinking method, we manufactured MEW-printed polycaprolactone (PCL) grids (Supplementary Fig. 24) and embedded them in HAMA hydrogels (2.0 w/v%). After UV-crosslinking, we selectively dissolved the PCL templates, and subjected the microchannel-containing HAMA constructs to shrinking (Fig. 3d). As revealed in Fig. 3e, the pristine MEW meshes embedded in the hydrogels were 20 ± 1 μm in diameter with an interfiber distance of 490 ± 29 μm. The microchannels created by sacrificing the MEW templates following washing and swelling were 39 ± 2 μm in diameter, and the distance between the adjacent microchannels (length of grid) was 772 ± 10 μm (Fig. 3f). After immersing in 2.0 w/v% HM_w_ chitosan in 1.0 v/v% acetic acid aqueous solution for 24 h, the microchannels shrank down to 10 ± 2 μm (~50.9% of original size, Fig. 3g, h), which was comparable to the diameter of single capillaries. In addition, the grid length was reduced to 292 ± 16 μm (~59.5% of the original size, Fig. 3g, i) with an associated decrease in the overall volume of the HAMA constructs. As such, using our shrinking method, the sacrificial MEW fibers of tens of micrometers could also be used to still generate sub-micron microchannels without needing the ultrafine fibers to start with.

### Shrinking behaviors of coaxially printed cannular constructs

Human tissues contain various cannular structures, such as the blood vessels for transporting cells, nutrients, and waste^26^, the lymphatic vessels for draining fluids^27^, and the tubules present in the kidney for secretion and reabsorption functions^28^. While sacrificial printing allows for emulation of matrix-embedded microchannels, microfluidic printing enabled by the adoption of coaxial, concentric printheads has featured single-step fabrication of standalone cannular structures. Both others^5,29^ and we^30,31^ have previously reported similar techniques in producing perfusable cannular tissues, where the minimum diameters of the obtained microfibers were limited to no smaller than a couple hundred micrometers due to the physical constraints of the sizes of the multilayered nozzles.

**Fig. 4:**
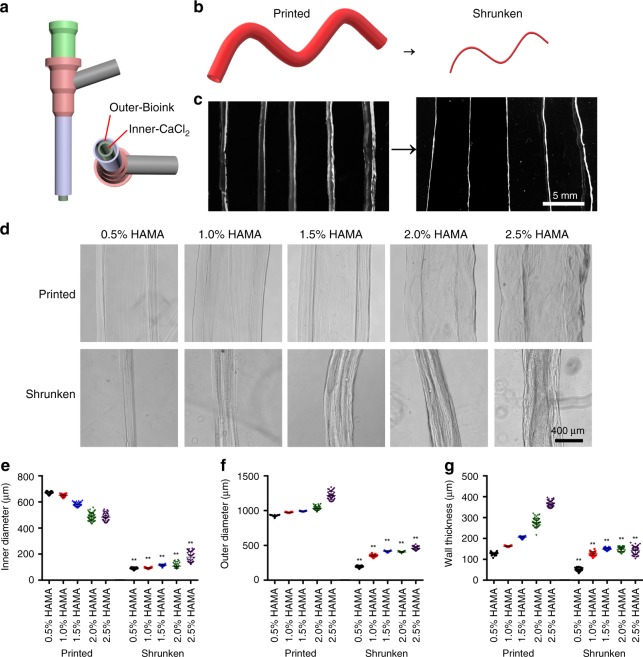
Coaxial printing of cannular HAMA-based constructs and their shrinking behaviors. **a** Schematic illustrations of the core-sheath coaxial nozzle used as the printhead, where the ink is delivered through the sheath flow and the CaCl_2_ solution is co-delivered through the core flow. **b** Printing of the cannular construct and its subsequent shrinkage. **c**, **d** Photographs and micrographs showing the size changes of the tubes, coaxial-printed with inks containing different concentrations of HAMA (0.5–2.5 w/v%), before and after 24 h of shrinkage in 2.0 w/v% HM_w_ chitosan dissolved in 1.0 v/v% acetic acid aqueous solution. **e**, **f**, **g** Corresponding quantitative analyses of diameter (**e**, inner diameter; **f**, outer diameter; **g**, wall thickness) changes before and after shrinkage. **P < 0.01; one-way ANOVA (**e**, **f**, **g**, compared with the corresponding as-printed structures); mean ± s.d. (*n* = 40).

A coaxial printhead was thus designed and optimized to print HAMA-based tubes (Fig. 4a, b). Different from direct extrusion printing and sacrificial printing, we mixed HAMA (0.5–2.5 w/v%) with alginate (0–2.0 w/v%) of different concentrations for use as the inks, to accommodate the microfluidic printing needs. The inks were delivered from the outer layer of the coaxial printhead, whereas the crosslinking CaCl_2_ solution (0.3 M) was carried from the interior. The printability of the inks was first examined (Supplementary Fig. 25). It was observed that, when the concentrations of HAMA ranged from 0.5 to 1.0 w/v% and the alginate was in the range of 0.5–2.0%, the tubes were smoothly printed and uniform in shape (Fig. 4c, d). The cannular structures became non-uniform when the concentration of HAMA was increased to higher than 1.0 w/v%. The optimal ink composition consisted of 1.0 w/v% HAMA, 0.5 w/v% alginate and 0.5 w/v% photoinitiator, and was used throughout the subsequent experiments.

The inner diameters of the as-printed tubular structures were measured at 670 ± 10 μm (0.5 w/v% HAMA), 648 ± 10 μm (1.0 w/v% HAMA), 583 ± 16 μm (1.5 w/v% HAMA), 478 ± 28 μm (2.0 w/v% HAMA), and 483 ± 29 μm (2.5 w/v% HAMA) (Fig. 4e–g). The tubes were then subjected to the same shrinking process (2.0 w/v% HM_w_ chitosan in 1.0 v/v% acetic acid aqueous solution for 24 h) we here established. After shrinking, the inner diameters narrowed down by 3–8 times of their original sizes to 90 ± 7 μm, 93 ± 4 μm (Supplementary Movie 2), 115 ± 7 μm, 108 ± 19 μm, and 176 ± 41 μm, respectively (Fig. 4e). Their outer diameters were decreased to 20.7–42.0% of original ones as well (Fig. 4f), so did the wall thicknesses (38.7–79.1%, Fig. 4g). We also compared the ratios obtained by the outer diameter divided by the inner diameter (OD/ID) before and after shrinkage, as well as the ratios of the outer diameter divided by the wall thickness (OD/WT), as measurements of fidelity of shrinkage in the printed cannular structures. As shown in Supplementary Fig. 26a, before shrinkage, the ratios of OD/ID were 1.38 ± 0.02 (0.5 w/v% HAMA), 1.50 ± 0.02 (1.0 w/v% HAMA), 1.71 ± 0.04 (1.5 w/v% HAMA), 2.16 ± 0.13 (2.0 w/v% HAMA), and 2.52 ± 0.03 (2.5 w/v% HAMA) for the respective tubes. After shrinkage, the ratios increased to 2.15 ± 0.22, 3.78 ± 0.26, 3.64 ± 0.23, 3.85 ± 0.60, and 2.73 ± 0.58, respectively. As for the OD/WT, there were decreases comparing to the as-printed cannular structures, from 7.25 ± 0.33, 5.97 ± 0.13, 4.83 ± 0.15, 3.74 ± 0.23, and 3.32 ± 0.03 to 3.81 ± 0.35, 2.73 ± 0.07, 2.76 ± 0.06, 2.73 ± 0.19, and 3.30 ± 0.47, respectively (Supplementary Fig. 26b). These metrics can guide the designs of cannular structures before shrinking according to the needs of the final desired sizes post-shrinking.

A printhead constructed with smaller needles (inner: 30G, outer: 18G) was used to print thinner HAMA/alginate tubes, which possessed an inner diameter, outer diameter, and wall thickness of 301 ± 3, 438 ± 6, and 68 ± 3 μm, respectively (Supplementary Fig. 27a, b). Following shrinking, the sizes were reduced to 37 ± 3, 148 ± 14, and 56 ± 7 μm, respectively. This set of values is close to the sizes of small blood vessels (arterioles and venules, 8–100 μm)^26^, lymphatic capillaries (30–80 μm)^27^, and proximal tubules (50–60 μm)^28,32^, making them physiologically relevant. The changes in the volumes and Young’s moduli of constructs formed with inks containing different concentrations of HAMA but constant 0.5 w/v% alginate before and after shrinking in 2.0 w/v% HM_w_ chitosan were measured (Supplementary Fig. 28), as well as for HM_w_ chitosan and Q. chitosan (both dissolved at 2.0 w/v % in 1.0 v/v % acetic acid aqueous solution) or in a perchloric acid solution of pH = 1 (Supplementary Fig. 29), and similar trends were found as reported for the HAMA hydrogels earlier.

### Shrinking bioprinting for applications involving cells

We endeavored on two aspects that are relevant to the future applications of this shrinking printing strategy in cell cultures, i.e., to expand its conceptual feasibility to a more bioactive ink of GelMA, and to prove the concept that the density of embedded cells in a sacrificially bioprinted construct may be increased through the shrinking process without significantly affecting their viability.

We first investigated whether our shrinking concept could be extended to GelMA, which is a gelatin derivative featuring intrinsic cell-binding moieties and is capable of on-demand photocrosslinking^9,33^. GelMA solutions or hydrogels also exhibit a net negative charge under neutral or slightly acidic pH values^34^, and we therefore hypothesized that an environment rich of cationic polymers would also shrink bioprinted GelMA constructs. Indeed, GelMA constructs sacrificially bioprinted to contain microchannels of 612 ± 32 μm in diameter through the fugitive Pluronic F127 ink shrank to 55.6 ± 9.1% of the original size (341 ± 56 μm) when immersed in 2.0 w/v% chitosan in 1.0 v/v% acetic acid aqueous solution (Supplementary Fig. 30a, b), and the distortions were deemed to be in an acceptable range (Supplementary Fig. 31).

To demonstrate the versatility of our shrinking technology, we further used a modified embedded sacrificial printing method^7^ to generate microchannels within GelMA/HAMA hydrogel constructs using gelatin as the fugitive ink, and subsequently illustrated their ability to be shrunken (using 2.0 w/v% HM_w_ chitosan dissolved in 1.0 v/v% acetic acid aqueous solution) and perfused (Supplementary Fig. 32, Supplementary Movies 3, 4). Consistent with other reports, it was shown that endothelial cells could be populated on the surfaces of the microchannels when seeded post-shrinking, indicating the reasonably good biocompatibility of the GelMA/HAMA-chitosan matrix after complexation (Supplementary Fig. 33). In addition, GelMA constructs made from MEW-PCL templates through washing out the PCL shrank from 19 ± 1 to 12 ± 2 μm, or ~62.1% of original size, whereas the length of grid was reduced from 503 ± 26 μm to 333 ± 16 μm, or ~66.3% of the original dimension (Supplementary Fig. 30c–g).

In microfluidic bioprinting, the inner diameter of the resultant GelMA (5.0 w/v%)/alginate (0.5 w/v%) cannular constructs decreased from 313 ± 8 μm prior to shrinking to 39 ± 4 μm afterwards (Supplementary Fig. 27c, d and Supplementary Movie 2), which was 12.5% of its original size or a factor 8 in reduction. The outer diameter and the wall thickness also became proportionally smaller. The shrinking results were comparable to those with bioprinted HAMA constructs.

**Fig. 5:**
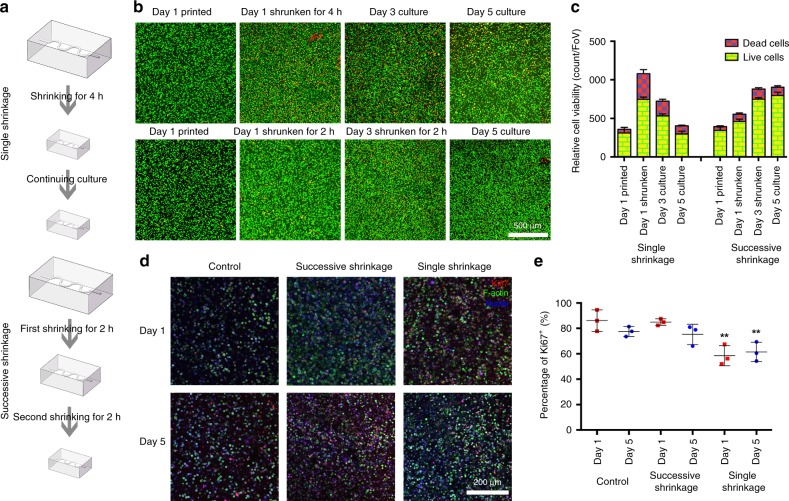
Biocompatibility of the shrinking process and shrinking in the presence of cells. **a** Schematics showing the single shrinkage (upper) and the successive shrinkage (lower). **b** Micrographs showing live (green)/dead (red) staining of MCF-7 cells embedded in GelMA/HAMA constructs following a single shrinking process (upper) and a successive shrinking process (lower). **c** Corresponding quantitative analyses of the numbers of live/dead cells during the two type of shrinking processes. **d** Fluorescence micrographs of MCF-7 cells in GelMA/HAMA constructs without any treatment (control) as well as after successive shrinkage and single shrinkage, stained for Ki67 (red), F-actin (green), and nuclei (blue). **e** Corresponding quantitative analyses of the percentages of Ki67^+^ stained nuclei in the three groups. **P < 0.01; one-way ANOVA (compared with the control group on Day 1), mean ± s.d. (**b**, **c** *n* = 10; **d**, **e** *n* = 1, d**e**viations obtained from eight distinct layers of a confocal stack for each sample).

We subsequently explored the cytocompatibility of the shrinking method, where the Q. chitosan solution at physiological pH was used as the shrinking agent. A mixture of GelMA (2.5 w/v%) and HAMA (0.5 w/v%) was adopted as the ink to improve the bioactivity of the hydrogel constructs over those made from pure HAMA. We designed two shrinking processes and compared their effects on size reduction and cell viability. The first procedure consisted of a single shrinkage step (Fig. 5a), i.e., a hydrogel construct encapsulating MCF-7 breast cancer cells, was left in 1.0 w/v% Q. chitosan in culture medium for 4 h, while in the second procedure termed successive shrinkage, we shrank the same type of construct twice in the same shrinking agent each time for 2 h, on the 1st day and the 3rd day (Fig. 5b). Through live/dead staining, we found that the cell density was significantly elevated after 4 h of shrinkage (single shrinkage; 1080 ± 49 total cells per field of view [FoV], i.e., 1417 by 1417 μm^2^), compared to shrinkage for only 2 h (549 ± 40 total cells per FoV), on the 1st day (Fig. 5b, c). In contrast, the original cell density was calculated to be in between 350 and 400 total cells per FoV. Nevertheless, a large number of cells were dead in the single-shrinkage process likely due to the prolonged exposure to the shrinking agent. On the 3rd day, the samples in the successive shrinkage group were shrunken again for another 2 h, which brought the cell density up to 883 ± 32 total cells per FoV. It should be noted that, although this density was lower than that of the single-shrinkage method on the 1st day, when we counted only viable cells, the values were in fact similar (748 ± 19 versus 747 ± 29 live cells per FoV), indicating that two successive size reductions each at a lower degree could minimize the harm of the process to the embedded cells and maintain their proliferation potential. Indeed, this trend became more pronounced during longer cultures. The viability of MCF-7 cells on the 5th day in the successive shrinking group (905 ± 49 total cells per FoV, percentage of live cells: 88.1 ± 4.7%, or 797 ± 42 live cells) was much higher than those in the single shrinkage group (406 ± 32 total cells per FoV, percentage of live cells: 73.8 ± 8.9%, or 297 ± 36 live cells). Ki67 was also assessed as a proliferation marker to show proliferation before and after shrinkage in GelMA/HAMA constructs. As revealed in Fig. 5d, e, the percentage of Ki67^+^ stained nuclei in the control group at the 1st day was 86.1 ± 6.5%, and for the cells in the successive shrinkage group, the positive rate was 84.9 ± 2.6%, similar to the control group. In contrast, it significantly decreased to 58.5 ± 8.0% when single shrinkage for 4 h was adopted. After 5 days of culture, the percentage of Ki67^+^ stained nuclei were 77.5 ± 4.0, 75.3 ± 8.0, and 61.4 ± 7.6% in the control group, successive shrinkage group, and single shrinkage group, respectively. Interestingly, the MCF-7 cells that underwent two successive shrinkages still exhibited a good proliferative potential comparable to the control group and higher than the single shrinkage group at the same time.

The method was extended to several other cell types including the C2C12 mouse skeletal muscle cells, which maintained satisfactory viability post-shrinking. As shown in Supplementary Fig. 34, the C2C12 cells spread well in the as-printed GelMA/HAMA hydrogel constructs, and after 2 h of shrinkage the density of the cells was doubled although their sizes seemed to have decreased possibly caused by the shrinking process. However, following 3 days of culture, the cells were able to spread again and proliferated throughout the subsequent culture period.

We noted that however, while MCF-7 cells and C2C12 cells performed reasonably well after shrinkage, another cell type that we examined, i.e., human umbilical vein endothelial cells (HUVECs), appeared to be much more sensitive to the shrinking processes. The percentage of Ki67^+^ stained nuclei was analyzed as a proliferation marker; at the 5th day of culture, only 42.2 ± 3.0% (successive shrinkage group) and 31.1 ± 2.1% (single shrinkage group) of HUVECs were Ki67^+^stained, significantly lower than the control group at the 1st day of culture (Supplementary Fig. 35a, b). We reason that such observations might be relevant to the differential sensitivities of the different cell types to the shrinking agent, Q. chitosan, for which we measured the metabolic activity of MCF-7 cells (Supplementary Fig. 36a) and HUVECs (Supplementary Fig. 36b) exposed to Q. chitosan PBS solutions at different concentrations for 30 min, 2, 4, and 24 h. The concentrations reflecting 50% reduction in cell metabolic activity, depicted as toxic concentration (TC)_50_ values of these two cell types were calculated. Indeed, as revealed by Supplementary Fig. 36, the TC_50_ values for MCF-7 cells at 30 min, 2, 4, and 24 h of Q. chitosan treatment were 0.505, 1.487, 0.371, and 0.131 mg mL^−1^, respectively, whereas those for HUVECs were significantly lower at all time points at 0.002, 0.077, 0.002, and 0.009 mg mL^−1^, respectively. These results suggested that HUVECs are remarkably more sensitive to Q. chitosan than MCF-7 cells, explaining their pronounced reduction in proliferation potential during the shrinking conditions that we used, even in the case of two successive shrinkage. We anticipate that these observations will provide insights towards selection of shrinking agent concentrations for sensitive and resistant cells in the future.

We finally demonstrated the feasibility of shrinking sacrificially printed hydrogels in the presence of cells. GelMA/HAMA constructs containing green fluorescent protein-labeled HUVECs (GFP-HUVECs) were produced with the Pluronic fugitive inks, and subjected to the two different shrinking procedures. As shown in Supplementary Fig. 37a, b, in the single shrinkage group, the microchannels inside the block were reduced to 64.2 ± 3.5% of their initial diameters. In the successive-shrinkage group, the microchannels shrank to 74.9 ± 5.8% after the first shrinkage, and after another shrinkage on the 3rd day, the diameter of the microchannels was reduced to 58.3 ± 7.3% of its original size, similar to the case of single shrinkage and within the physiological range of small blood vessels. As expected, the loss of the GFP signals was more pronounced in the single shrinkage group.

## Discussion

In conclusion, we report here a printing strategy of complexation-induced resolution enhancement, i.e., shrinking printing, through post-treatment of the printed structures without changing the printer hardware or printing parameters. We conducted our proof-of-concept studies with several techniques of printing and succeeded in all cases, including direct extrusion printing, sacrificial printing, and microfluidic hollow tube printing. Notably, our data showed that these printed constructs could reduce in their sizes by different degrees, comparing to their original dimensions. In addition, results indicated that this method is broadly applicable, i.e., a printed anionic hydrogel structure might be shrunken by a cationic polymer, or vice versa. We finally demonstrated that successive shrinking could preserve, in a cell type-dependent manner, the viability of cells embedded in the printed hydrogel matrices compared to a single, longer shrinking procedure, revealing the potential applications of our shrinking printing method towards tissue biofabrication. We therefore anticipate widespread adoption of our unique technology in future printing of hydrogel constructs for various application areas with further optimizations.

## Methods

### Synthesis and characterizations of HAMA

Hyaluronic acid was functionalized with methacrylate groups through a transesterification reaction with methacrylic anhydride^8,35^. In a typical synthesis, 3 g of hyaluronic acid sodium salt (*M*_w_: 1530 kDa, Lifecore Biomedical, USA) was dissolved overnight in 400 mL of de-ionized water at 4 °C. The solution was placed on ice and an equal volume of dimethyl formamide (DMF, Sigma-Aldrich, USA) was added whilst stirring vigorously. Methacrylic anhydride (MA, Sigma-Aldrich) was added in a 5:1 molar ratio of MA (5.8 g, 37.5 mmol) to hyaluronic acid disaccharide units over the course of 4 h using a syringe pump (1.375 mL h^−1^). During these 4 h, the pH was controlled using a Mettler DL21 titrator (Mettler-Toledo, The Netherlands) connected to a pH meter, which dispensed an aqueous 0.5M NaOH (Sigma-Aldrich) solution whenever the pH of the solution dropped below 8.5. After complete addition of the methacrylic anhydride, pH was monitored for an additional hour and maintained above pH 8.5. Subsequently, the reaction mixture was left at 4 °C overnight. The next day, NaCl (Sigma-Aldrich) was dissolved in the reaction mixture to up to 0.5 M and the mixture was precipitated in 10 equal volumes of ethanol at −78 °C (cooled with an acetone dry ice bath). HAMA was collected as dry white pellets, dissolved in de-ionized water and dialyzed against de-ionized water for 2 days to remove impurities (Visking, regenerated cellulose dialysis membrane, molecular weight cut off [MWCO]: 12–14 kDa, VWR, The Netherlands). After dialysis the HAMA solution was freeze-dried to yield a white powder.

### Synthesis and characterizations of FITC-Q. chitosan

Quaternized chitosan was fluorescently labeled using FITC (Sigma-Aldrich). In short, 1 g of Q. chitosan was dissolved overnight in 200 mL of freshly made sodium carbonate buffer (pH = 9.0). Subsequently, a fresh solution of 10 mg of FITC in 10 mL of dry dimethylsulfoxide (DMSO, Sigma-Aldrich) was added dropwise to the Q. chitosan solution under vigorous stirring. The reaction was left to proceed for 4 h. Both the dissolution FITC in DMSO solution and the reaction with Q. Chitosan were performed in a dark environment to limit potential photodegradation. Upon completion of the reaction, the solution was directly poured into dialysis bags (MWCO: 10–14 kDa) and dialyzed in de-ionized water for 6 days whilst protected from light. Finally, the dialyzed solution was freeze-dried overnight to yield FITC-Q. chitosan as an orange powder.

The degree of substitution of FITC onto the Q. chitosan was studied using UV-vis spectrometry, where absorbances were measured at 280 (A_280_) and 488 (A_488_) nm, respectively. Molar extinction coefficients of 175 and 68 M cm^−1^ were used for Q. Chitosan (ε_c_) and FITC (ε_FITC_), respectively. Labeling density was determined according to:

$${\mathrm{Q.Chitosan}}\,{\mathrm{concentration}} \, (M) = \frac{{A_{280} - (A_{488} \times CF)}}{{\varepsilon _C}}$$

and

$${\mathrm{Moles}}\,{\mathrm{of}}\,{\mathrm{FITC}}\,{\mathrm{per}}\,{\mathrm{mol}}\,{\mathrm{Q.Chitosan}} = \frac{{A_{488}}}{{\varepsilon _{{\mathrm{FITC}}} \times {\mathrm{Q.Chitosan}}\,{\mathrm{concentration}}}}$$

It was found that one mole of Q. chitosan was conjugated with 29.7 mol of FITC.

### Synthesis and characterizations of GelMA

GelMA was synthesized by reaction of type B bovine skin gelatin (~225 bloom, Sigma-Aldrich) with methacrylate anhydride at 50 °C for 1 h in PBS (pH = 7.4, Gibco, USA)^36^. Methacrylic anhydride was added dropwise in a 0.6:1 weight ratio of anhydride to gelatin. Next, the solution was diluted 1:1 with de-ionized water and dialyzed for 5 days (dialysis membrane as used for the HAMA synthesis), and subsequently freeze-dried to yield a white powder.

### Determination of degree of methacrylation and methacrylate conversion

The degree of methacrylation (DM) for the synthesized HAMA was determined using a previously developed high-performance liquid chromatography (HPLC) method^37^. In short, 15 mg of polymer or dried hydrogel was dissolved overnight at room temperature in 6 mL of aqueous 0.02-M NaOH solution. Next, 1 mL of acetic acid was added and the samples were injected into an Alliance Waters HPLC system equipped with UV-VIS detection monitoring at 210 nm (Dual Lambda Absorbance, USA) and a Sunfire C18 column (column temperature: 50 °C). An isocratic method was used based on eluent consisting of 15:85 acetonitrile: de-ionized water (pH = 2, adjusted with perchloric acid) with a set flow of 1 mL min^−1^. The samples were referenced to a calibration curve of known concentrations of methacrylic acid. Concentrations were then calculated to yield the DM, defined as the number of methacrylate groups per 100 disaccharide units. The DM of HAMA was found to be 28.8 ± 0.4% (*n* = 3).

The DM of GelMA was defined as the number of methacrylate groups per available lysine found in the gelatin and was determined by ^1^H NMR in D_2_O according to Hoch et al.^38^. The DM of GelMA was found to be approximately 50%.

### Preparation and printability of the HAMA inks

For the inks used in extrusion printing and sacrificial printing, various concentrations (w/v) of HAMA (0.5%, 1.0%, 1.5%, 2.0%, or 2.5%) were dissolved in de-ionized water in room temperature overnight. In addition, 0.5 w/v% photoinitiator (Irgacure 2959; Sigma-Aldrich) was added to initiate photocrosslinking upon UV-irradiation (approximately 10 mW cm^−2^, 360–480 nm, 40 s). For the blend inks for coaxial printing, different concentrations of HAMA (0–3.0 w/v%) and alginate (0–2.0 w/v%; lot number: BCBP9590V, Sigma-Aldrich) were evaluated, where the final formulations were determined to be 0.5, 1.0, 1.5, 2.0, or 2.5 w/v% HAMA + 0.5 w/v% alginate + 0.5 w/v% photoinitiator. Inks were prepared and stored at 4 °C until use.

### Preparation of GelMA inks

For the inks used in sacrificial printing, the GelMA was dissolved in de-ionized water in 37 °C for 1 h at a concentration of 5.0 w/v%. In addition, 0.5 w/v% photoinitiator was added to enable crosslinking. In the blend ink used for coaxial printing, the final formulation was 5.0 w/v% GelMA + 0.5 w/v% alginate + 0.5 w/v% photoinitiator. Inks were stored at 4 °C until use.

### Preparation of chitosan solutions and shrinking efficiency in different chitosan solutions

Four types of chitosan were used, where three of different molecular weights (LM_w_: 15 kDa; MM_w_: 50–190 kDa; HM_w_: 700–800 kDa) were of similar degree of deacetylation (85%) and from Golden-Shell, China. Q. chitosan (*M*_w_: 50–100 kDa), which was ~90% deacetylated and subsequently 95% quaternized to yield permanent, was from Cool Chemistry, China. They were dissolved at a concentration of 2.0 w/v%, in 1.0 v/v% acetic acid aqueous solution when comparing the effect of chitosan of different molecular weights and types. Another three types of chitosan with different degrees of deacetylation (72.5%, 77.8%, and 94.6%; Heppe Biomedical, Germany) were used to study the effects of shrinking agent deacetylation. All chitosan types were of similar molecular weights (50–250 kDa), and were all dissolved at a concentration of 2.0 w/v% in 1.0 v/v% acetic acid aqueous solution for usage. 1.0 w/v% HAMA hydrogels were fabricated and immersed in these shrinking agents for 24 h and the volumes before and after shrinking were measured.

Various concentrations (0.5, 1.0, 1.5, 2.0, 3.0, or 5.0 w/v%) of HM_w_ chitosan and Q. chitosan were dissolved in 1.0 v/v% acetic acid aqueous solution, de-ionized water, or DMEM (Gibco) at 37 °C. The solutions were vortexed and stored at 4 °C. Before use, they were pre-heated to 37 °C. The printed hexagons were immersed in HM_w_ chitosan (0.5, 1.0, 1.5, 2.0, 3.0, or 5.0 w/v%) dissolved in 1.0 v/v% acetic acid aqueous solution, or Q. chitosan (0.5, 1.0, 1.5, 2.0, 3.0, or 5.0 w/v%), which was dissolved in 1.0 v/v% acetic acid aqueous solution, de-ionized water, or DMEM. The shrinking processes were recorded at 2 and 24 h microscopically and through photography, and measurements were made using imageJ (National Institutes of Health, USA).

### Preparation of hydrogel discs and dimension measurements

The anionic polymer solutions (2.0 w/v% HAMA, 2.0 w/v% alginate) were cast in circular PDMS molds (D = 4.5 cm) and subsequently gelled through either exposure to UV irradiation (HAMA) or a CaCl_2_ solution (alginate), or both. Hydrogels that were gelled with CaCl_2_ were washed with de-ionized water to remove excess ions. Subsequently, biopsy punches (Integra Miltex, The Netherlands) were used to produce cylindrical hydrogels (*D* = 6 mm, *H* = 2 mm, ~56.5 µL or *D* = 8 mm, *H* = 3.4 mm, ~170.9 µL).

The cationic polymer (HM_w_ chitosan) solution was prepared in 1.0 v/v% acetic acid in water at concentrations of 2.0 w/v%. The HM_w_ chitosan solution was then cast into the PDMS molds, and subsequently, 2 mL of glutaraldehyde (with concentrations of 200, 400, 800, or 2400 µM) in 1.0 v/v% acetic acid solution was gently pipetted on top. The crosslinking reaction was left to proceed for 1 h, biopsy punch was used to produce cylindrical hydrogels (*D* = 6 mm, *H* = 2 mm, ~56.5 µL). The hydrogels were incubated in a perchloric acid solution (pH = 1.0), 1.0 v/v% acetic acid aqueous solution (pH = 4.7), or 1.0 v/v% acetic acid aqueous solution with 2.0 w/v% of the shrinking agent. For all hydrogel shrinking studies, measurements before and after shrinking were performed using Vernier calipers (measurement error ~30 µm).

### Measurements of mechanical properties

To measure the Young’s modulus and volume of HAMA hydrogels with/without alginate before and after shrinkage, crosslinked hydrogel disks (*D* = 6 mm, *H* = 2 mm, ~56.5 µL) were prepared as mentioned before, incubated for 24 h in HM_w_ chitosan, Q. chitosan (both dissolved up to 2.0 w/v% in 1.0 v/v% acetic acid aqueous solution), or an aqueous solution of perchloric acid adjusted to pH 1.0. Moreover, the hydrogels containing alginate were briefly incubated in a CaCl_2_ solution before shrinking. Compression tests were performed in triplicate on a 2980 DMA (TA Instruments, the Netherlands) with a ramp of 2.0 N min^−1^ up to a maximum of 18.0 N. The elastic modulus was calculated as the slope of the start of the stress-strain curve that was obtained from the compression test. Specifically, we used the linear region between 10 and 30% strain.

### Stability studies of the shrunken hydrogels

The following hydrogel formulations were used to study the stability of the shrinking effect, i.e., 2.0 w/v% HAMA, 1.5 w/v% HAMA + 0.5 w/v% alginate, and 0.5 w/v% HAMA + 2.0 w/v% GelMA. All hydrogels were incubated in 2.0 w/v% FITC-Q. chitosan solution in PBS until fully shrunken and then rinsed twice with PBS. The hydrogels were weighed and measured (diameters and heights), and then incubated at 37 °C in individual vials containing 1 mL of PBS per vial. The PBS was completely changed every 2 day. The absorbance (*λ* = 488 nm) of the supernatant was determined on Days 1, 3, 7, 11, 15, and 21. In addition, the diameters and heights of the hydrogels were also measured at these time points. After all measurements, the hydrogels were placed back into the vials and fresh PBS was added. By measuring the absorbance of a standard curve of known FITC concentrations at 488 nm and through factoring in the degree of FITC labeling of the Q. chitosan, the amounts of FITC-labeled Q. chitosan released (µg mL^−1^) from the hydrogels per time point were calculated.

### SEM sample preparation and imaging

Hydrogels consisting of 1.0 w/v% HAMA were fabricated and incubated in 2.0 w/v% HM_w_ chitosan in 1.0 v/v% acetic acid aqueous solution for 24 h and freeze-dried, or freeze-dried immediately post-fabrication. The freeze-dried samples were cut using a razor blade, and subsequently sputter-coated with a nanometer-layer of Pt. The sputter-coated samples were imaged with SEM (Phenom^TM^, FEI, The Netherlands). An electron beam of 5 kV was used, and the samples were imaged at 1000 times of magnification.

### Extrusion printing

Constructs were first designed by 3D Studio Max (Autodesk, USA) and sliced by Repetier (Hot-World, Germany). An Allevi 2 bioprinter (Allevi, USA) was used to fabricate the constructs. For the extrusion printing of hexagonal patterns, the printhead moving speed was 6 mm s^−1^, and the inks were crosslinked by exposing to UV light (~10 mW cm^−2^, 360–480 nm, 40 s). In this case, we did not use in situ photocrosslinking for our printing processes but performed post-printing photocrosslinking. The HAMA inks were sufficiently viscous to maintain the shape stability immediately post-printing. The printed and photocrosslinked HAMA hexagons were immersed in the chitosan solutions (0.5, 1.0, 1.5, 2.0, 3.0 or 5.0 w/v% in 1.0 v/v% acetic acid aqueous solution) for 24 h. We used microscopy (Eclipse Ti, Nikon, Japan) and a camera (Canon, Japan) to image the constructs after 2 h and 24 h of incubation. We also measured the printability and the Young’s modulus of HAMA constructs made with different HAMA concentrations before and after shrinkage.

The alginate and chitosan hexagons were printed in the same way, where alginate hexagons were crosslinked in 0.3-M CaCl_2_ and chitosan hexagons crosslinked with glutaraldehyde (800 µM).

For the pyramid (six 10-mm lines converging in four vertices) hydrogel fabrication, the construct was designed and sliced by the same software as mentioned above. The ink consisted of 2.0 w/v% HAMA, 0.1 w/v% Irgacure 2959, and fluorescent microbeads (purple, 15-35 μm; CREATEX, USA), where a 1.5 w/v% gelatin type A (Sigma-Aldrich) hydrogel bath (formed by cooling at 4 ° C for 40 min) was used as the support matrix to facilitate freeform printing of the pyramid^8^. The structure was printed by an Allevi 2 bioprinter equipped with a 23G needle (BD Biosciences, USA), followed by crosslinking by exposing to UV light (~10 mW cm^−2^, 360–480 nm) for 60 s. After photocrosslinking, the gelatin bath was heated and stepwise replaced with water. Finally, the 3D-printed pyramidal structure was incubated in 1.0 w/v% HM_w_ chitosan in 1.0 v/v% acetic acid aqueous solution for 24 h for complete shrinkage.

### Sacrificial printing

Sacrificial printing based on Pluronic F127 followed previously established protocols^22^. Pluronic F127 (Sigma-Aldrich) solution was used as the fugitive ink in sacrificial printing, which is a hydrogel at room temperature but liquefies at low temperatures. Specifically, 40 w/v% Pluronic F127 aqueous solution was used for printing the fugitive templates. A PDMS mold (length: 1.5 cm, width: 0.5 cm) was first made. The 2.0 w/v% HAMA solution was cast into the PDMS mold at a thickness of 0.2 cm and UV-crosslinked (10 mW cm^−2^, 360–480 nm, 40 s) to act as the base layer. Then, Pluronic F127 (40.0 w/v%) mixed with fluorescent microbeads (red) was printed onto the HAMA gel surface. Subsequently, another layer of 2.0 w/v% HAMA solution was poured into the mold to cover the Pluronic, immediately followed by another UV crosslinking procedure. The construct was placed in water overnight at 4 °C to liquefy and remove the Pluronic, leaving the open channel. Later, the block with open channel was immersed in a HM_w_ chitosan solution (2.0 w/v% in 1.0 v/v% acetic acid aqueous solution) for 24 h. The diameter change of the channel was recorded before and after shrinking. Sacrificial printing of GelMA constructs was done in a similar fashion as for HAMA. It should be noted that, the Pluronic fugitive ink was not completely removed and the residual coating on the channels containing the fluorescent microbeads facilitated visualization.

Sacrificial printing in combination with an embedded freeform printing strategy was also demonstrated, where a blend solution of GelMA (2.5 w/v%) and HAMA (0.5 w/v%) containing 0.1 w/v% photoinitiator was cast into a PDMS mold and placed at 4 °C for 30 min until it became a semi-solid hydrogel bath. Then, 5.0 w/v% gelatin type A was prepared as the bioink at room temperature and printed directly into the GelMA/HAMA bath as a serpentine microfiber, then UV-crosslinked (10 mW cm^−2^, 360–480 nm, 60 s). The gelatin was subsequently washed out by incubating the block at 37 °C to form the microchannel. To shrink, the blocks with open channels were immersed in the HM_w_ chitosan solution (2.0 w/v% in 1.0 v/v% acetic acid aqueous solution) for 24 h. HUVECs were subsequently seeded into the microchannels post-shrinking. Perfusion of the channels before and after shrinking was also demonstrated.

Alternatively, PCL meshes were fabricated by the MEW technique and used as sacrificial templates^25^ and 2.0 w/v% HAMA was cast around the templates. To leach the PCL template from the HAMA constructs, a multi-stage removal process was optimized. The constructs were immersed sequentially in de-ionized water for 1.5 h, 50 v/v% acetone (Sigma-Aldrich) in water for 1.5 h, in 100 v/v% acetone overnight, 50 v/v% dichloromethane (DCM, Sigma-Aldrich) in acetone for 1.5 h, and 100 v/v% DCM overnight. After dissolution of the PCL, the hydrogel was treated in the reverse order of the steps described until in 100% de-ionized water for rehydration. Later, the block with open channels was immersed into the chitosan solution (2.0 w/v% in 1.0 v/v% acetic acid aqueous solution) for 24 h. The diameters of channels and the lengths of grids were recorded and analyzed.

### Coaxial printing

A coaxial printhead containing two injection channels was fabricated, where the size of the internal needle was 23G and the outer was 16G. These two needles were fixed concentrically with epoxy resin (Devcon, USA). The internal channel was perfused with a 0.3-M CaCl_2_ solution (Sigma-Aldrich) and the external channel was used to print blend inks of HAMA (0.5, 1.0, 1.5, 2.0, or 2.5 w/v%) + 0.5 w/v% alginate + 0.5 w/v% photoinitiator. Both layers of flows were controlled by syringe pumps (NE-1000, New Era Pump Systems Inc, USA) and the extrusion rates were set at 400 μL min^−1^ (0.3-M CaCl_2_ solution) and 200 μL min^−1^ (inks). The CaCl_2_ solution was used for immediate physical crosslinking of the alginate component for tube formation during printing, whereas the tubes were subsequently shrunken in the HM_w_ chitosan solution (2.0 w/v% in 1.0 v/v% acetic acid aqueous solution) for 24 h and UV-crosslinked (~10 mW cm^−2^, 360–480 nm, 40 s). The ID, OD, WT, as well as the ratios of OD/ID and OD/WT before and after shrinkage were all measured. Alternatively, a coaxial printhead made of 30 G/18 G needles was also produced to print smaller-sized tubes using the blend ink containing 1.0 w/v% HAMA or 5.0 w/v% GelMA, together with 0.5 w/v% alginate and 0.5 w/v% photoinitiator.

### Visualization of shrinking distortions using non-rigid registration

The distortions of co-registered pre-shrinking/2-h shrinking structures and post-shrinking structures were visualized by deforming the pre-shrinking/2-h shrinking images using a non-rigid registration process to attempt an exact match of the post-shrinking images of the corresponding samples^18^. Using a B-spline based non-registration algorithm (i), which generates a deformation grid between the pre-shrinking/2-h shrinking (green) and post-shrinking (magenta) patterns, we were able to map the deformation between them. Pre-shrinking/2-h shrinking and post-shrinking images were first converted to binary images using the Matlab function im2bw (ii), which converts an image to a binary image, based on threshold, by replacing all the pixels in the input image with luminance greater than the level 1 (white) and replacing all other pixels with the value 0 (black). Both images were smoothened for faster registration using a Gaussian blur filter, with a standard deviation of 5 pixels.

(i) D.-J. Kroon, “B-spline Grid, Image and Point based Registration”; available at: www.mathworks.com/matlabcentral/fileexchange/20057-b-spline-grid–image-and-point-based-registration.
(ii) Matlab Documentation, “im2bw”; available at: https://www.mathworks.com/help/images/ref/im2bw.html.

### Shrinking bioprinting in the presence of cells

MCF-7 breast cancer cells (American type culture collection [ATCC], USA) were suspended in 2.5 w/v% GelMA + 0.5 w/v% HAMA aqueous solutions at a density of 1.0 × 10^7^ cells/mL, 0.3 w/v% photoinitiator (lithium phenyl-2,4,6-trimethylbenzoylphosphinate, LAP; Allevi) was added for inducing photocrosslinking (~10 mW cm^−2^, 360–480 nm, 20 s). Half of the samples were shrunken once for 4 h (single shrinkage, the 1st day) in 1.0 w/v% Q. chitosan in DMEM supplemented with 10 v/v% fetal bovine serum (FBS, Gibco), or shrunken twice at 2 h each (successive shrinkage, the 1st day and the 3rd day). In the single shrinkage group, live/dead staining was performed before and after shrinking on the 1st day, the 3rd day, and the 5th day. For the successive shrinkage group, the live/dead staining was carried out before shrinkage and subsequently on the 1st day, the 3rd day, and the 5th day. The specimens with cells were rinsed with PBS and incubated with 2 μM of calcein-AM and 4 μM of ethidium homodimer-1 (Invitrogen, USA) for 30 min to examine viability. Moreover, the cells were also stained for Ki67 as a proliferation marker. The samples were fixed in 4% paraformaldehyde (Thermo Fisher, USA) for 15 min, permeabilized with 0.05% Triton X-100, and then blocked with 2% FBS and 2% bovine serum albumin (BSA, Sigma-Aldrich) in PBS. Samples were incubated with recombinant anti-Ki67 antibody conjugated to Alexa Fluor® 594 (Abcam, USA) overnight at 4 °C. FITC-phalloidin (Cytoskeleton, USA) was used to stain for F-actin and the nuclei were counter-stained with 4′,6-diamidino-2-phenylindole (DAPI, Vector Laboratories, USA). The samples were then rinsed in PBS and visualized using confocal laser scanning microscopy (LSM880, Zeiss, Germany) and measurements were made using image J. The same protocols were used for shrinking HUVECs (ATCC)-encapsulated hydrogel constructs and associated analyses, only the cultures were maintained in endothelial cell growth medium (EGM-2, PromoCell, USA) and the constructs were shrunken in 1.0 w/v% Q. chitosan in EGM-2. C2C12 mouse skeletal myoblasts (ATCC) were also encapsulated in the same GelMA/HAMA hydrogel and evaluated against the shrinking process. The samples were fixed in 4% paraformaldehyde for 15 min, permeabilized with 0.05% Triton X-100, and then blocked with 2% FBS and 2% bovine serum albumin in PBS. FITC-phalloidin was used to stain F-actin and the nuclei were counter-stained with DAPI. The samples were visualized by an inverted fluorescence microscope (Eclipse Ti).

For the IC_50_ experiments, MCF-7 cells and HUVECs were cultured in 96-well plates until confluency. The cells were exposed to different concentrations (0.5 μg mL^−1^–0.5 mg mL^−1^) of Q. chitosan dissolved in culture medium for various lengths of time (30 min–24 h). After exposure, the cells were incubated with the PrestoBlue^TM^ Cell Viability Reagent (Thermo Fisher) in medium for 30 min and measured for absorbance.

We further tested the shrinkage of sacrificially bioprinted GelMA/HAMA constructs in which GFP-HUVECs (Angio-Proteomie, USA) were encapsulated within the hydrogels where each construct contained a central microchannel formed by removing the Pluronic template embedded with red fluorescent microbeads. The procedure was similar to that without cells, but the Pluronic was washed out in PBS after liquefying at 4 °C for 5 min. Before and after shrinking in 1.0 w/v% Q. chitosan in EGM-2. The samples were visualized with the inverted fluorescence microscope (Eclipse Ti).

### Statistical analysis

Data are presented as the mean ± standard deviation (SD). Differences between the values were evaluated using one-way analysis of variance (ANOVA; Tukey’s post hoc test) or two-tailed Student’s *t*-test. Data are presented as means with 95% confidence interval. *p* < 0.05 was considered statistically significant.

## Supplementary information

Supplementary Information

Description of Additional Supplementary Files

Supplementary Movie 1

Supplementary Movie 2

Supplementary Movie 3

Supplementary Movie 4

## Data Availability

The datasets that support the findings of this study are available from the corresponding authors upon reasonable request. All requests for raw and analyzed data and materials will be promptly reviewed by the Brigham and Women’s Hospital and Utrecht University to verify whether the request is subject to any intellectual property or confidentiality obligations. Any data and materials that can be shared will be released via a Material Transfer Agreement.

## Code availability

The codes developed in this study are available from the corresponding authors upon reasonable request. All requests will be promptly reviewed by the Brigham and Women’s Hospital and Utrecht University to verify whether the request is subject to any intellectual property or confidentiality obligations. Any codes that can be shared will be released via a Material Transfer Agreement.
