# Supplementary material for: Complexation-induced resolution enhancement of 3D-printed hydrogel constructs
Source: Nat Commun. 2020 Mar 9;11:1267. doi: 10.1038/s41467-020-14997-4 (PMC7062888; doi:10.1038/s41467-020-14997-4)
Supplement: Supplementary file 1 — Supplementary Information [file 41467_2020_14997_MOESM1_ESM.pdf]

# Supplementary Information for "Complexation-Induced Resolution Enhancement of 3D-Printed Hydrogel Constructs"

**Gong et al.**

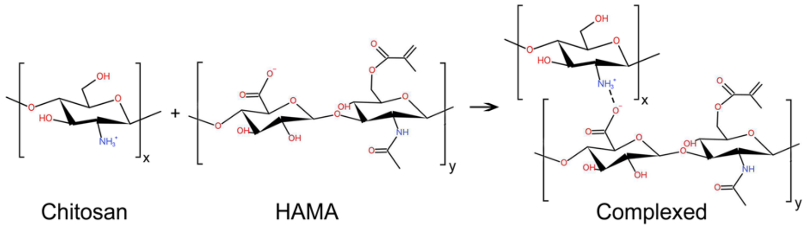


**Supplementary Figure 1.**

**Chemical structures of HAMA, chitosan, and their charge complexation interaction.** The primary amine groups on chitosan and the carboxyl groups on HAMA undergo a complexation interaction, followed by water expulsion from the hydrogel, which forms the basis of the shrinking behavior of the HAMA hydrogels.

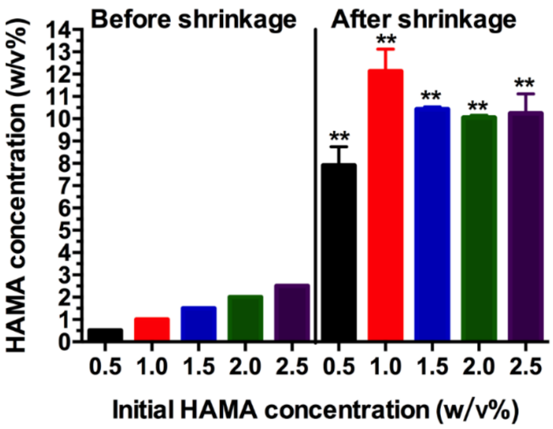


**Supplementary Figure 2.**

Estimated changes of concentrations of HAMA in the hydrogels made with different initial HAMA concentrations (left) before and (right) after shrinkage in 2.0 w/v% $HM_w$ chitosan dissolved in 1.0 v/v% acetic acid aqueous solution for 24 h. **$P < 0.01$; one-way ANOVA (compared with the values of corresponding as-prepared samples); mean ± SD (n = 3).

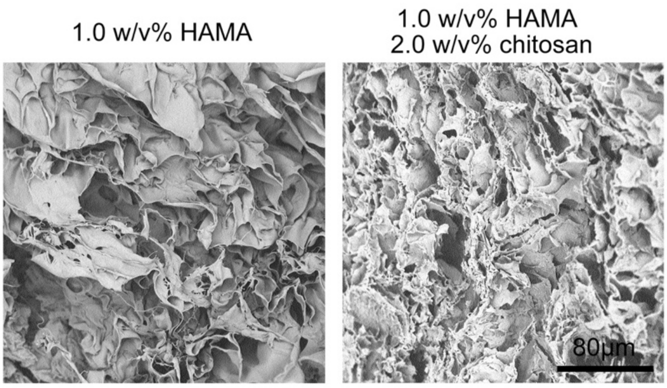


**Supplementary Figure 3.**

SEM images of HAMA hydrogels before and after shrinkage, for 1.0 w/v% HAMA hydrogel constructs (left) before shrinkage and (right) shrunken in 2.0 w/v% $HM_w$ chitosan for 24 h.

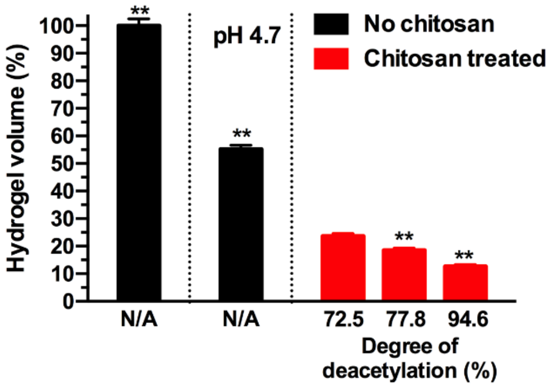


**Supplementary Figure 4.**

Changes in volumes of 1.0 w/v% HAMA hydrogel discs as-prepared, shrunken in 1.0 v/v% acetic acid aqueous solutions (pH = 4.7) or 2.0 w/v% chitosan of different deacetylations in 1.0 v/v% acetic acid aqueous solutions (pH = 4.7). $^{**}P < 0.01$; one-way ANOVA (compared with the values of samples in deacetylation = 72.5% group); mean ± s.d.% (n = 3).

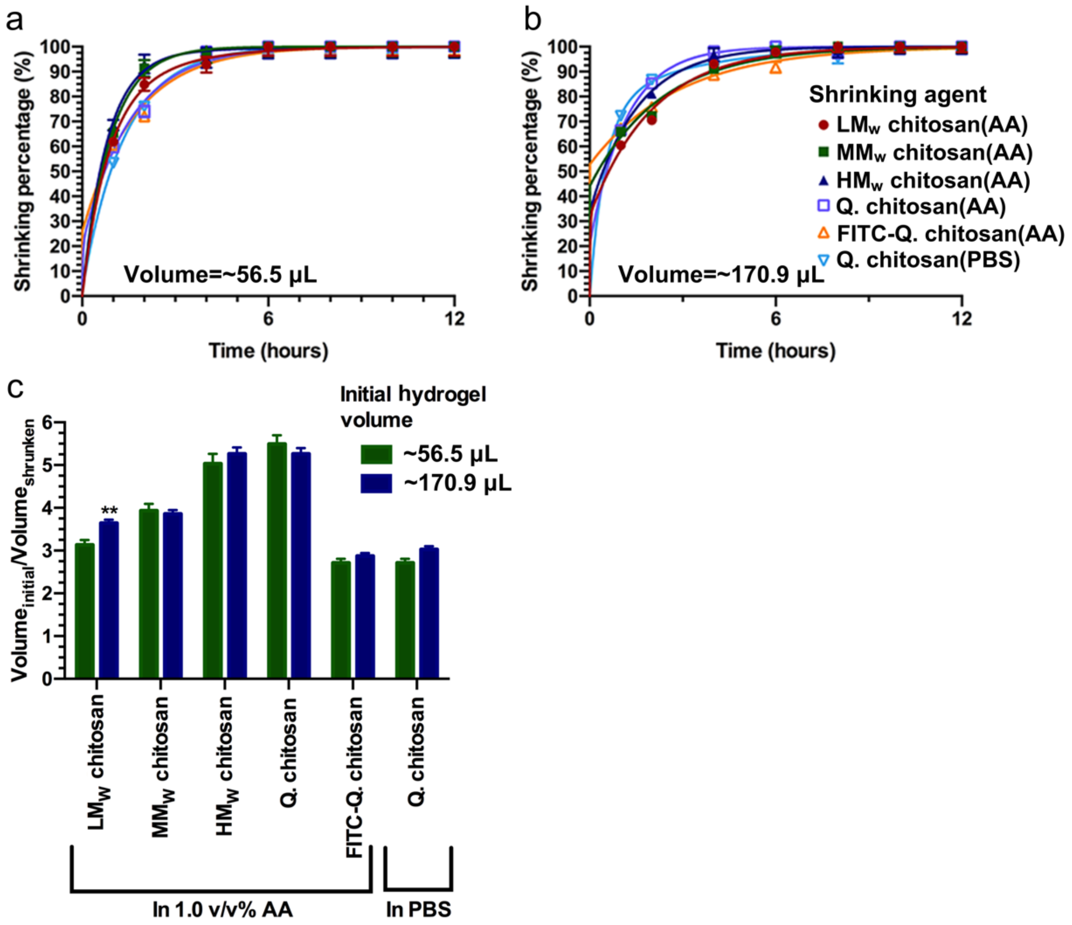


**Supplementary Figure 5.**

Shrinking kinetics of 2.0 w/v% HAMA hydrogels of two different volumes (**a**, ~56.5 μL and **b**, ~170.9 μL), in 2.0 w/v% chitosan of different molecular weights and types in 1.0 v/v% acetic acid aqueous solution (AA) or in PBS. **c**, Corresponding quantitative analyzes of the shrinkage ratio (initial volume/shrunken volume) in 2.0 w/v% chitosan of different molecular weights and types in 1.0 v/v% acetic acid aqueous solution or in PBS, $^{**}P < 0.01$; two-tailed Student's *t*-test (**c**, compared with the hydrogel of ~56.5μL in initial volume); mean ± s.d. (n = 3).

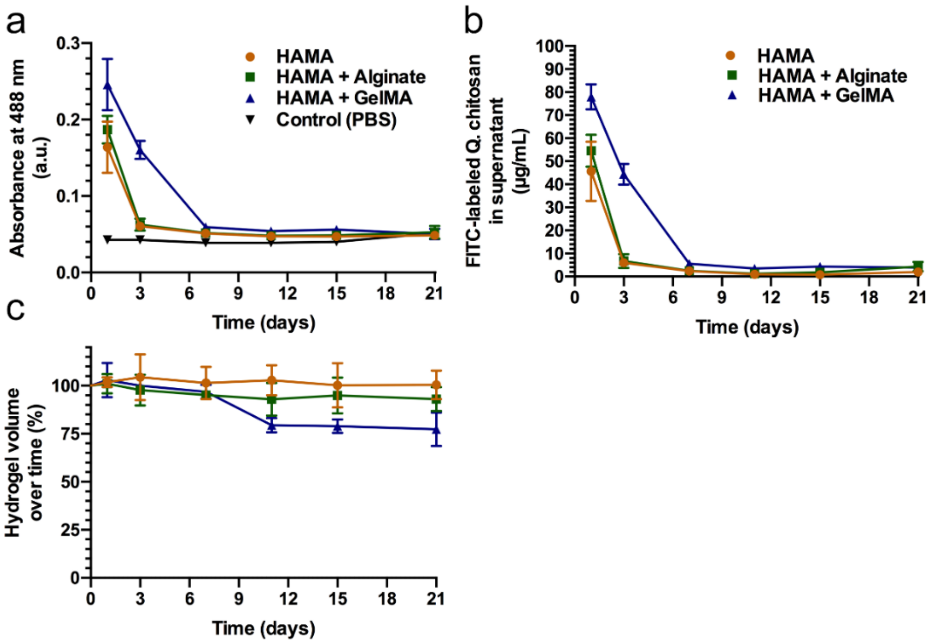


**Supplementary Figure 6.**

**Stability of shrunken hydrogels. a**, Quantitative analyses of absorbance ($\lambda = 488$ nm) of the supernatant from fully shrunken hydrogels in PBS as a function of time, made of 2.0 w/v% HAMA, 1.5 w/v% HAMA + 0.5 w/v% alginate, and 0.5 w/v% HAMA + 2.0 w/v% GelMA, and the control group (PBS). **b**, Quantitative analyses of FITC-labeled Q. chitosan in the supernatant from fully shrunken hydrogels in PBS as a function of time, made of 2.0 w/v% HAMA, 1.5 w/v% HAMA + 0.5 w/v% alginate, and 0.5 w/v% HAMA + 2.0 w/v% GelMA. **c**, Quantitative analyses of volume changes of fully shrunken hydrogels in PBS as a function of time, made of 2.0 w/v% HAMA, 1.5 w/v% HAMA + 0.5 w/v% alginate, and 0.5 w/v% HAMA + 2.0 w/v% GelMA. mean ± s.d. (n = 5).

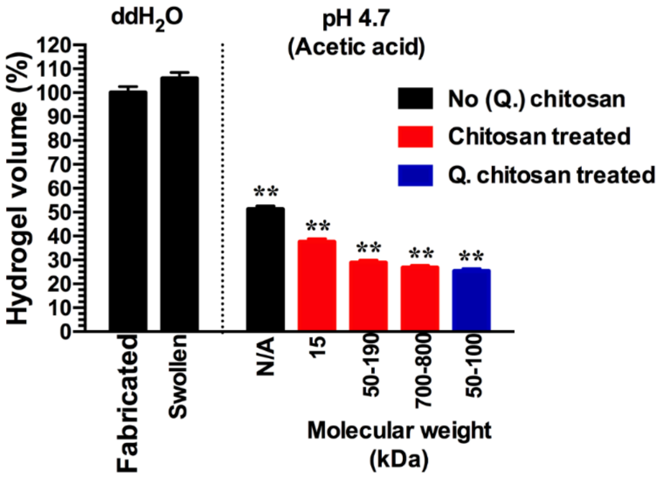


**Supplementary Figure 7.**

Changes in volumes of alginate hydrogel discs as-prepared, swollen in de-ionized water, or shrunken in 2.0 w/v% chitosan of different molecular weights and types in 1.0 v/v% acetic acid aqueous solutions (pH = 4.7). $^{**}P < 0.01$; one-way ANOVA (compared with the values of corresponding as-prepared samples); mean ± s.d. (n = 3).

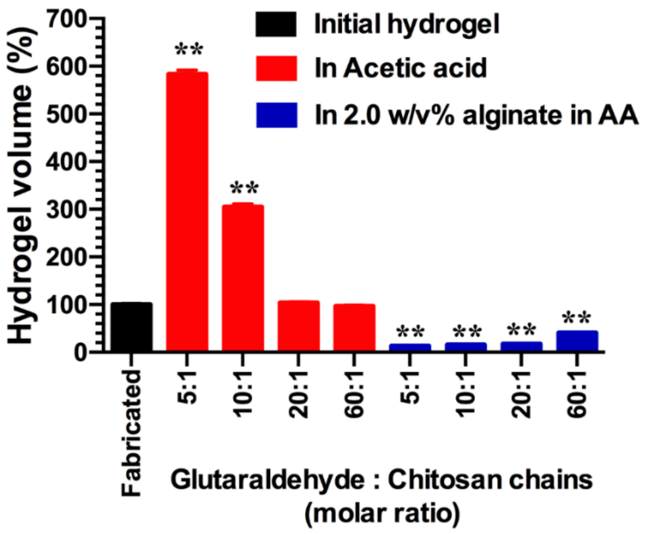


**Supplementary Figure 8.**

Quantitative analyses of 2.0 w/v% $HM_w$ chitosan hydrogels swollen and shrunken under different conditions with or without 2.0 w/v% alginate in 1.0 v/v% acetic acid aqueous solution. ** $P < 0.01$; one-way ANOVA (compared with the values of corresponding as-prepared samples); mean ± s.d. (n = 3).

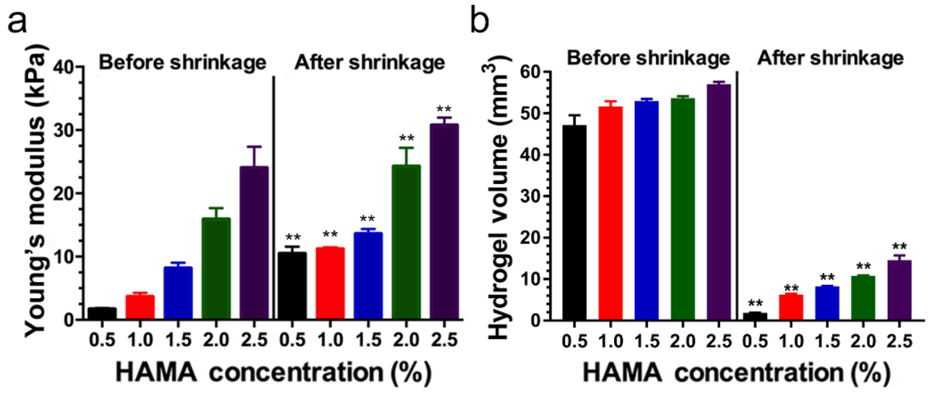


**Supplementary Figure 9.**

**Changes in Young's modulus and volume of HAMA hydrogel constructs. a**, Change in Young's modulus of HAMA hydrogels made with different HAMA concentrations before and after shrinking in 2.0 w/v% $HM_w$ chitosan. **b**, Change in volume of HAMA hydrogels made with different HAMA concentrations before and after shrinking in 2.0 w/v% $HM_w$ chitosan. $^{**}P < 0.01$; one-way ANOVA (compared with the values of corresponding as-prepared samples); mean ± s.d. (n = 3 or 4).

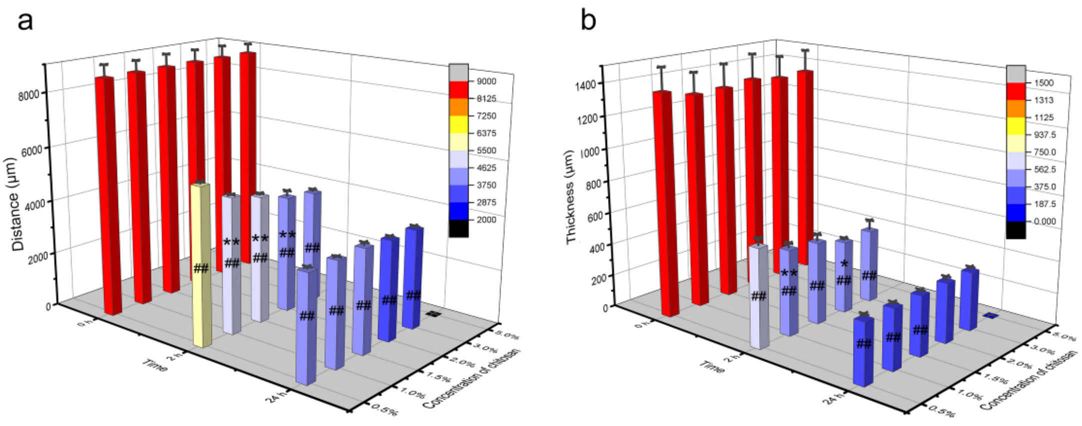


**Supplementary Figure 10.**

Quantitative analyses of (**a**) side-to-side distance and (**b**) thickness changes of extrusion-printed HAMA constructs (2.0 w/v%) following shrinking in different concentrations of $HM_w$ chitosan dissolved in 1.0 v/v% acetic acid aqueous solution. *$P < 0.05$, **$P < 0.01$; one-way ANOVA (compared with the group shrunken in chitosan solution of the immediate lower concentration); $^{\#\#}P < 0.01$; one-way ANOVA (compared with the preceding time point in the same group); mean ± s.d. (n = 10).

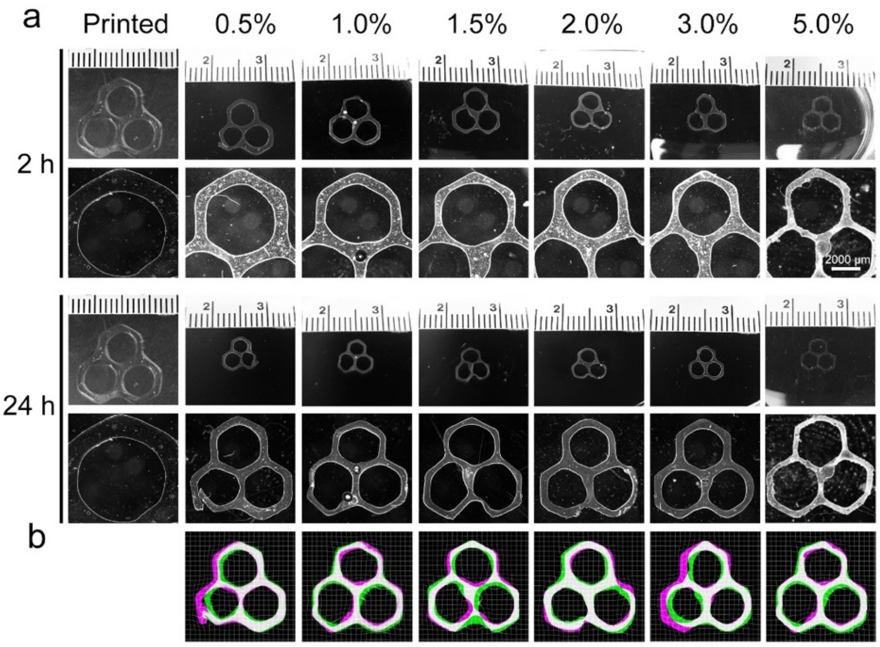


**Supplementary Figure 11.**

**a**, Photographs (upper) and micrographs (lower) showing size changes of printed HAMA hexagons (2.0 w/v%) at 2 h and 24 h of shrinking in different concentrations of Q. chitosan (0.5 - 5.0 w/v%) dissolved in 1.0 v/v% acetic acid aqueous solution. **b**, Vector-field maps comparing the 24 h-shrinking images (magenta) to the corresponding 2 h-shrinking images (green) by a B-spline-based non-rigid registration algorithm, where the overlaps appear in white and the grids show local distortions. Note that the 2 h-shrinking images have been rescaled to match the sizes of the 24 h-shrinking images to enable comparisons.

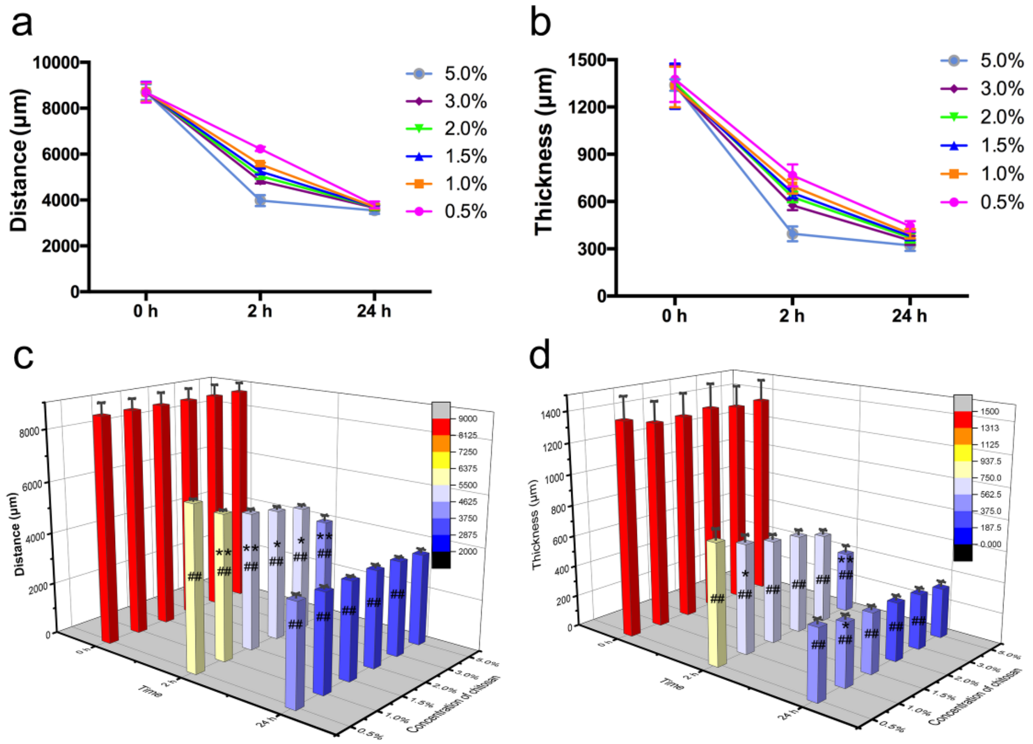


**Supplementary Figure 12.**

Quantitative analyses of (**a**, **c**) side-to-side distance and (**b**, **d**) thickness changes of extrusion-printed HAMA constructs (2.0 w/v%) following shrinking in different concentrations of Q. chitosan (0.5 - 5.0 w/v%) dissolved in 1.0 v/v% acetic acid aqueous solution. $^{*}P < 0.05$, $^{**}P < 0.01$; one-way ANOVA (**c**, **d**, compared with the group shrunken in Q. chitosan solution of the immediate lower concentration); $^{\#\#}P < 0.01$; one-way ANOVA (**c**, **d**, compared with the preceding time point in the same group); mean ± s.d. (n = 10).

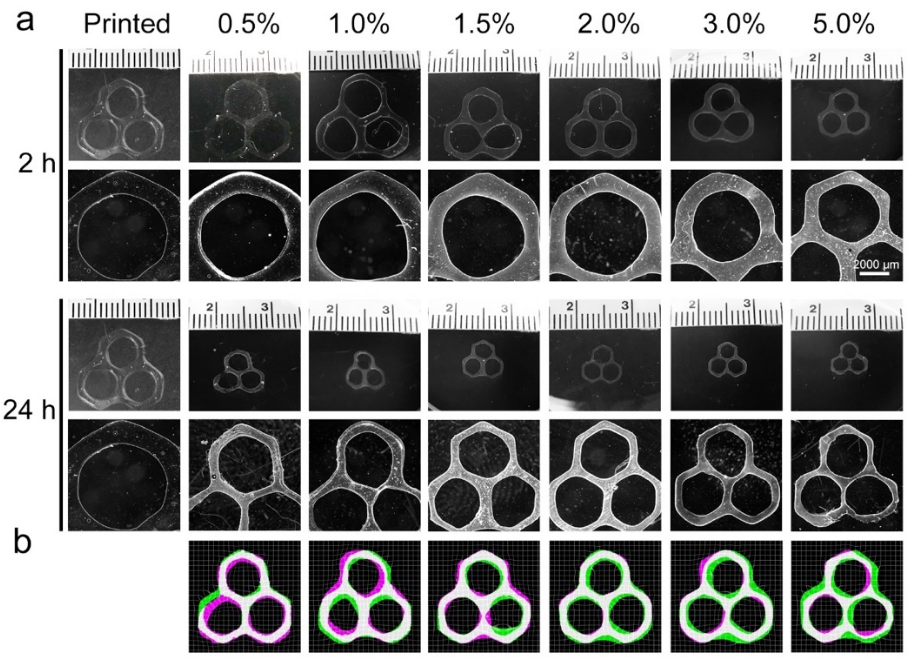


**Supplementary Figure 13.**

**a**, Photographs (upper) and micrographs (lower) showing size changes of printed HAMA hexagons (2.0 w/v%) at 2 h and 24 h of shrinking in different concentrations of Q. chitosan (0.5 - 5.0 w/v%) dissolved in de-ionized water. **b**, Vector-field maps comparing the 24 h-shrinking images (magenta) to the corresponding 2 h-shrinking images (green) by a B-spline-based non-rigid registration algorithm, where the overlaps appear in white and the grids show local distortions. Note that the 2 h-shrinking images have been rescaled to match the sizes of the 24 h-shrinking images to enable comparisons.

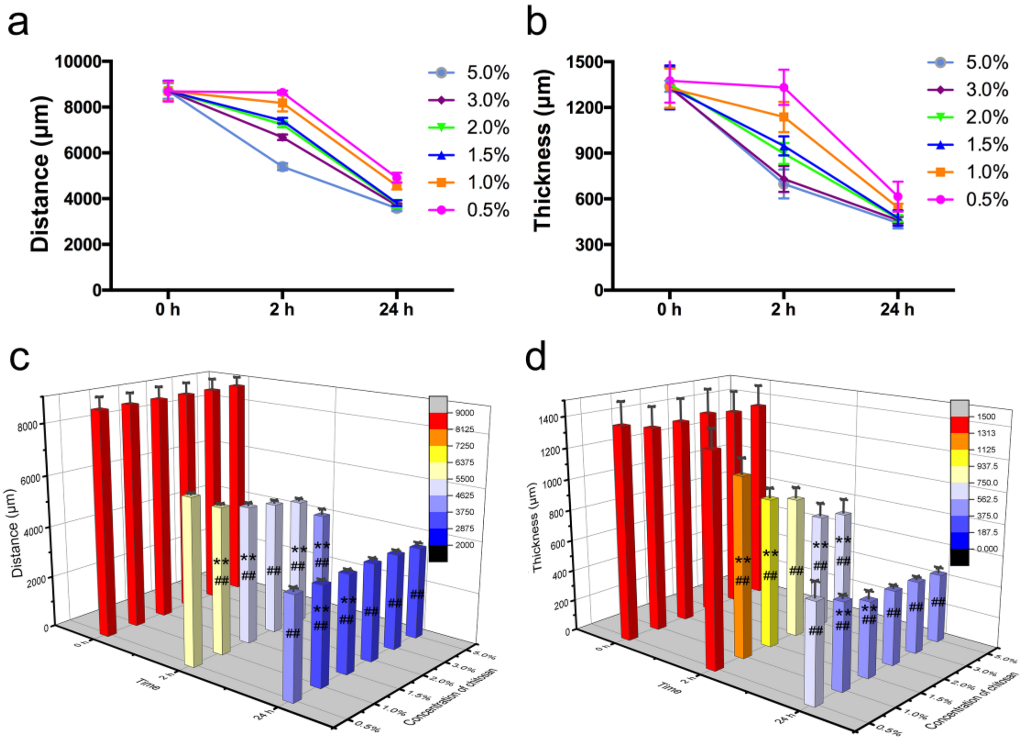


**Supplementary Figure 14.**

Quantitative analyses of (**a**, **c**) side-to-side distance and (**b**, **d**) thickness changes of extrusion-printed HAMA constructs (2.0 w/v%) following shrinking in different concentrations of Q. chitosan (0.5 - 5.0 w/v%) dissolved de-ionized water. $^{**}P < 0.01$; one-way ANOVA (**c**, **d**, compared with the group shrunken in Q. chitosan solution of the immediate lower concentration); $^{\#\#}P < 0.01$; one-way ANOVA (**c**, **d**, compared with the preceding time point in the same group); mean ± s.d. (n = 10).

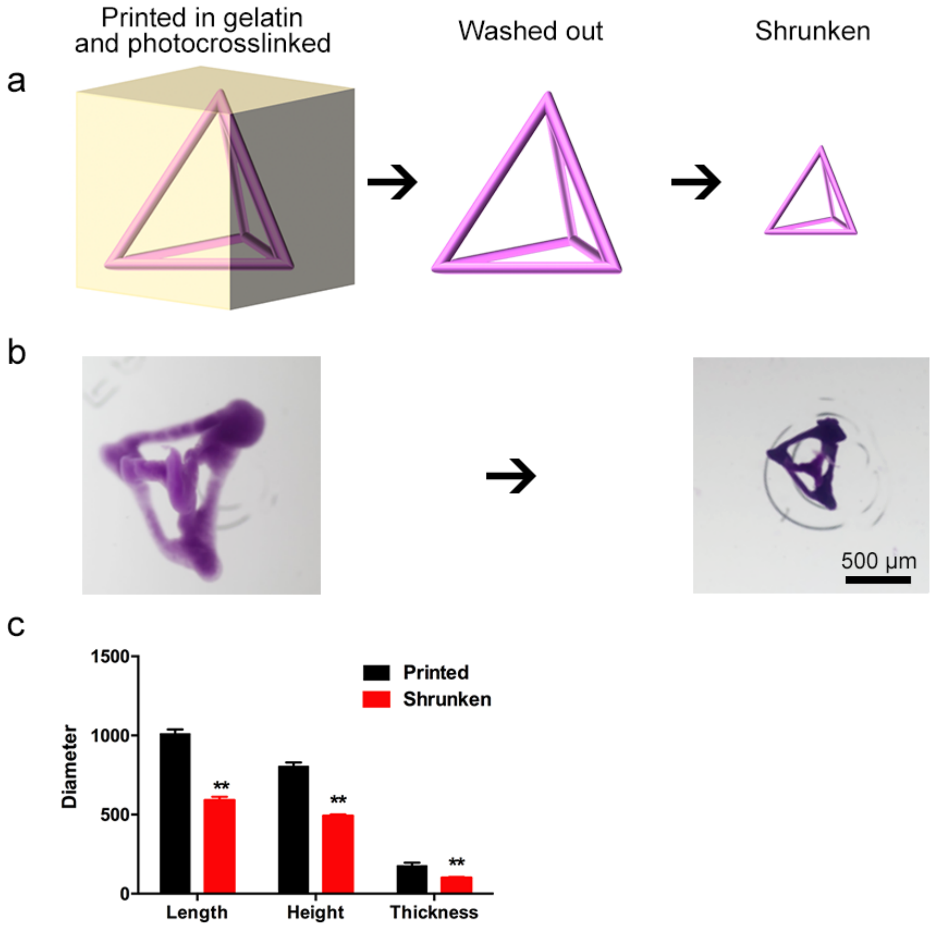


**Supplementary Figure 15.**

**a**, Schematic representation of the printing and photocrosslinking, washing, and shrinking process of a 3D structure. **b**, Photographs showing a printed and photocrosslinked HAMA (2.0 w/v%) pyramid in gelatin (1.5 w/v%, left), fully shrunken pyramid in 1.0 w/v% $HM_w$ chitosan dissolved in 1.0 v/v% acetic acid aqueous solution for 24 h (right). **c**, Quantitative analyses of length, height and thickness changes of extrusion-printed HAMA pyramid following shrinking in 1.0 w/v% $HM_w$ chitosan solution in 1.0 v/v% acetic acid aqueous solution. $^{**}P < 0.01$; two-tailed Student's t-test (compared with the corresponding as-printed structures); mean ± s.d. (n = 3).

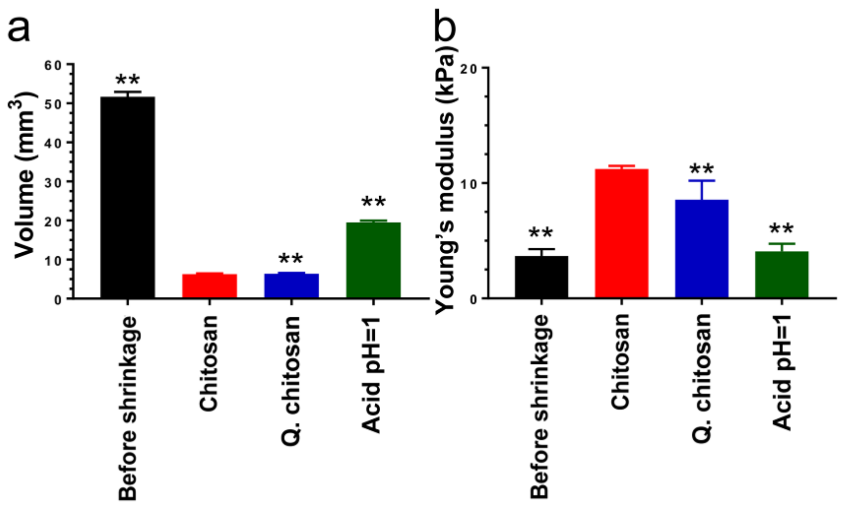


**Supplementary Figure 16.**

**Changes in volume and Young's modulus of HAMA hydrogel constructs. a**, Changes in volume of 2.0 w/v% HAMA hydrogels before and after shrinkage in $HM_w$ chitosan, Q. chitosan (both dissolved at 2.0 w/v % in 1.0 v/v % acetic acid aqueous solution), and an aqueous solution of perchloric acid at pH 1.0. **b**, Change in Young's modulus of 2.0 w/v% HAMA hydrogels before and after shrinkage in $HM_w$ chitosan, Q. chitosan (both dissolved at 2.0 w/v % in 1.0 v/v % acetic acid aqueous solution), and an aqueous solution of perchloric acid at pH 1.0. $^{**}P < 0.01$; one-way ANOVA (compared with the group shrunken in chitosan); mean ± s.d. (n = 3 or 4).

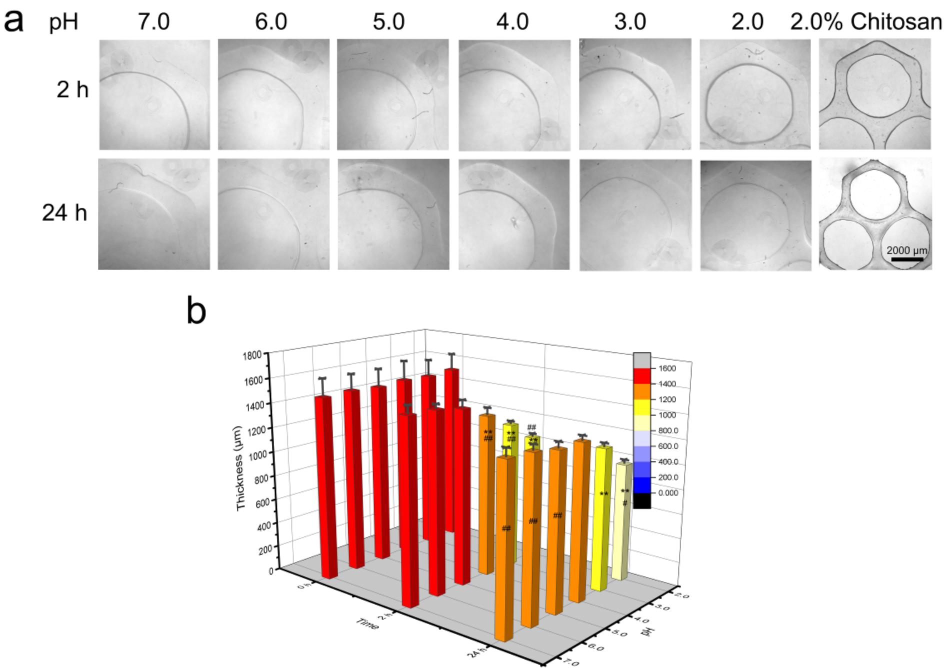


**Supplementary Figure 17.**

**a**, Micrographs showing size changes of printed HAMA hexagons (2.0 w/v%) at 2 h and 24 h of incubation in acetic acid aqueous solutions of different pH values (adjusted using acetic acid). The construct shrunken in 2.0 w/v% $HM_w$ chitosan dissolved in 1.0 v/v% acetic acid aqueous solution was used as the control. **b**, Corresponding quantitative analyses. $^{**}P < 0.01$; one-way ANOVA (compared with the group pH = 7); $^{\#}P < 0.05$, $^{\#\#}P < 0.01$; one-way ANOVA (compared with the preceding time point at the same pH); mean ± s.d. (n = 10).

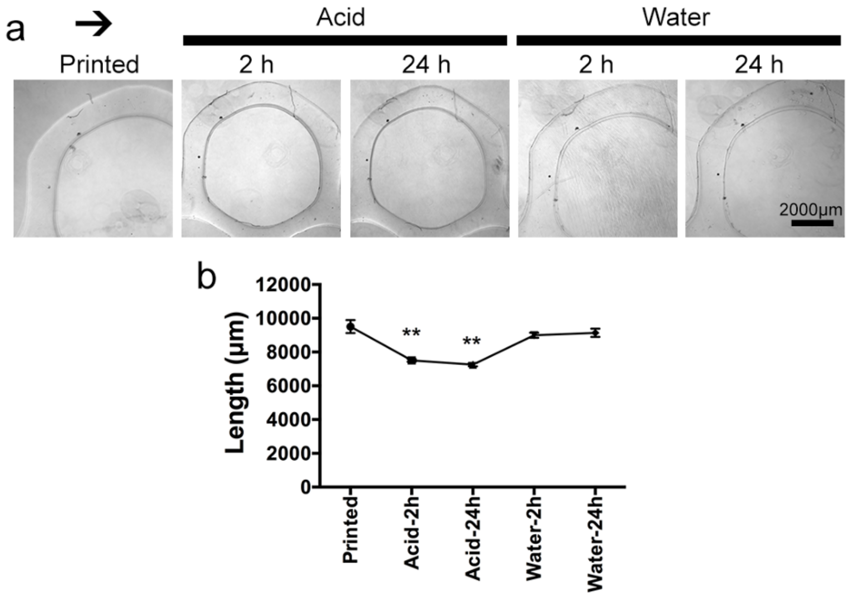


**Supplementary Figure 18.**

**Size change in acid and water. a**, Micrographs showing size change of a printed HAMA construct (2.0 w/v%) at 2 h and 24 h of incubation in acetic acid aqueous solutions at pH = 2, followed by incubation again in water for another 24 h. **b**, Corresponding quantitative analyses of the change in the side-to-side distance. ***P* < 0.01; one-way ANOVA (compared with the as-printed structures); mean ± s.d. (n = 10).

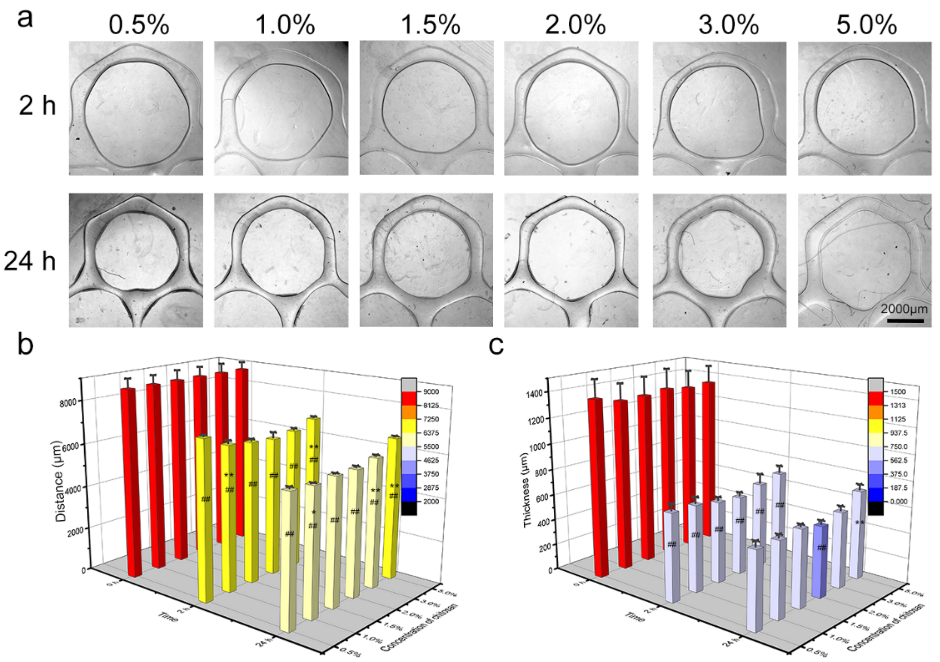


**Supplementary Figure 19.**

**a**, Micrographs showing size changes of printed HAMA hexagons (2.0 w/v%) at 2 h and 24 h of shrinking in different concentrations of Q. chitosan (0.5 - 5.0 w/v%) dissolved in DMEM. **b**, **c**, Quantitative analyses of (**b**) side-to-side distance and (**c**) thickness changes of extrusion-printed HAMA constructs (2.0 w/v%) following shrinking in different concentrations of chitosan dissolved in DMEM. *$P < 0.05$, **$P < 0.01$; one-way ANOVA (**b**, **c**, compared with the group shrunken in chitosan solution of the immediate lower concentration); $^{\#}P < 0.05$, $^{\#\#}P < 0.01$; one-way ANOVA (**b**, **c**, compared with the preceding time point in the same group of concentration); mean ± s.d. (n = 10).

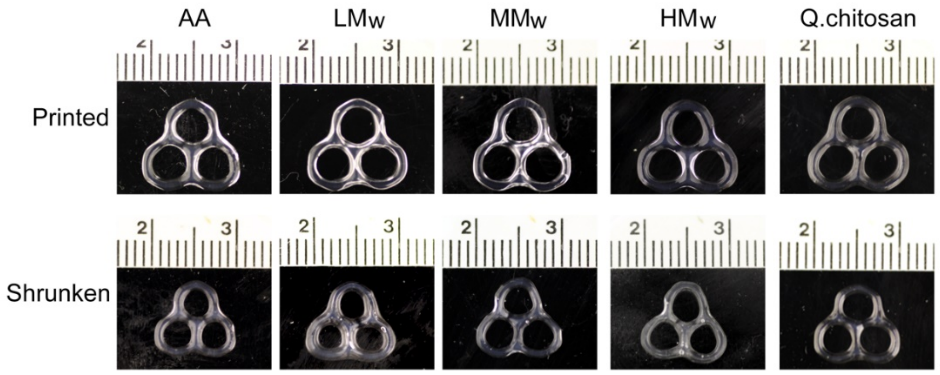


**Supplementary Figure 20.**

Micrographs showing size changes of printed and crosslinked 2.0 w/v% alginate hexagons before and after shrinkage in 1.0 v/v% acetic acid aqueous solution, or in 2.0 w/v% chitosan of different molecular weights and types in 1.0 v/v% acetic acid aqueous solutions.

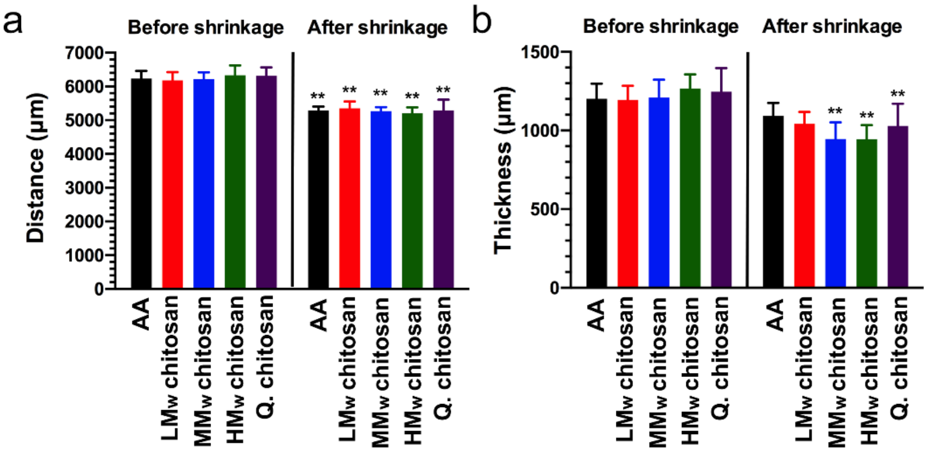


**Supplementary Figure 21.**

Corresponding quantitative analyses of size changes of the printed 2.0 w/v% alginate hexagons, in 1.0 v/v% acetic acid aqueous solution, or in 2.0 w/v% chitosan of different molecular weights and types in 1.0 v/v% acetic acid aqueous solutions, for (**a**) side-to-side distance and (**b**) thickness. $^{**}P < 0.01$; one-way ANOVA (compared with the corresponding as-printed structures); mean ± s.d. (n = 6).

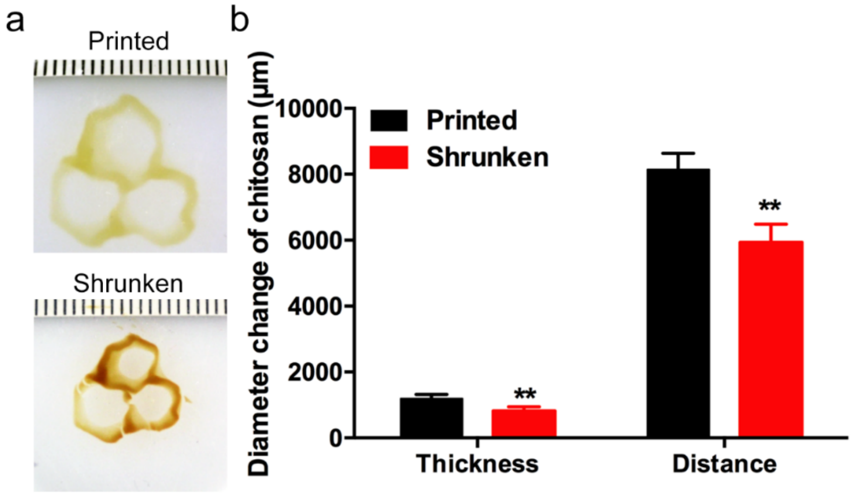


**Supplementary Figure 22.**

**a**, Photographs showing size changes of printed chitosan hexagons (2.5 w/v%, crosslinked by 800-μM glutaraldehyde) before and after shrinkage in 2.0 w/v% alginate solution in 1.0 v/v% acetic acid aqueous solution. **b**, Quantitative analyses of thickness and side-to-side distance changes of extrusion-printed chitosan constructs following shrinking in 2.0 w/v% alginate solution in 1.0 v/v% acetic acid aqueous solution. $^{**}P < 0.01$; two-tailed Student's t-test (compared with the corresponding as-printed structures); mean ± s.d. (n = 10).

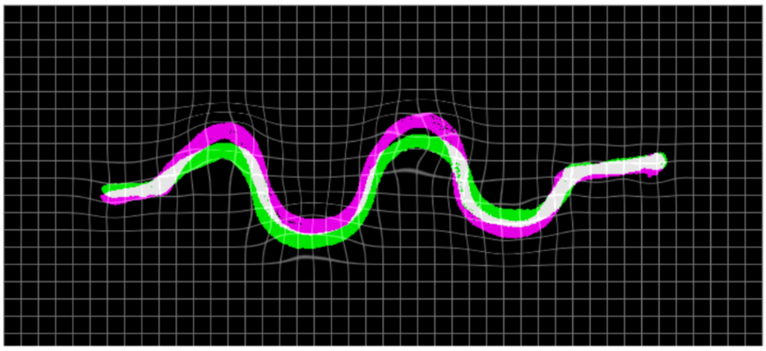

**Supplementary Figure 23.**

Vector-field map of the sacrificially printed microchannel-embedded HAMA hydrogel (relating to **Fig. 3b**), comparing the post-shrinking image (magenta) to the corresponding pre-shrinking image (green) by a B-spline-based non-rigid registration algorithm, where the overlaps appear in white and the grids show local distortions. Note that the pre-shrinking images have been rescaled to match the sizes of the post-shrinking images to enable comparisons.

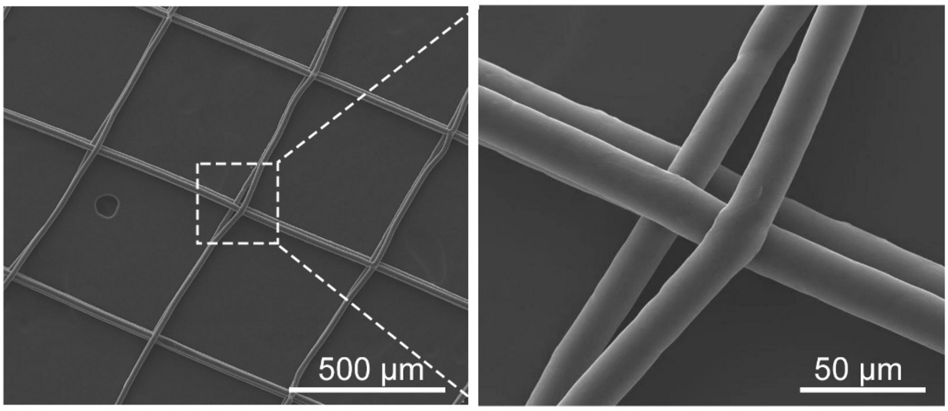


**Supplementary Figure 24.**

SEM images of a PCL mesh printed with MEW.

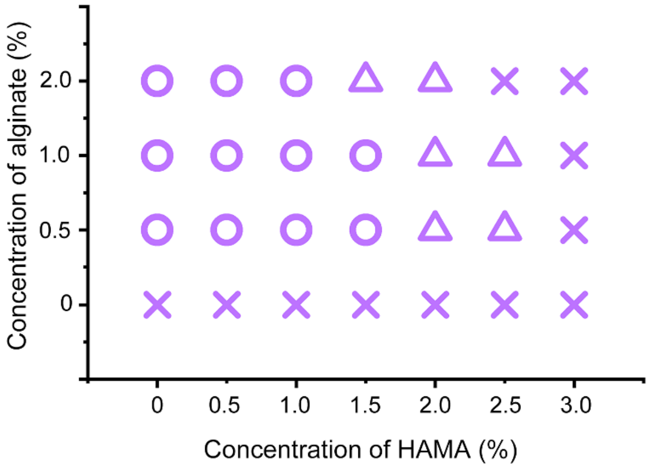


**Supplementary Figure 25.**

Printability mapping of HAMA/alginate ink of different concentrations of the components. ○: printable; △: partially printable but not uniform; x: non-printable.

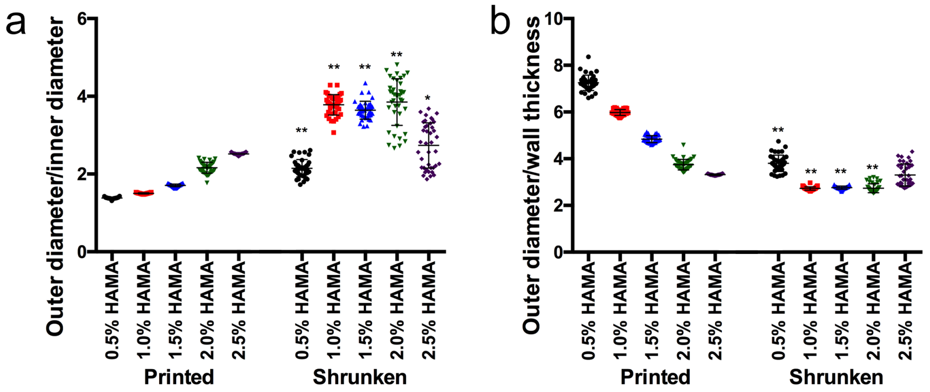


**Supplementary Figure 26.**

Quantitative analyses of the ratios of (**a**) OD/ID and (**b**) OD/WT before and after shrinkage, for printed tubular HAMA/alginate structures (relating to **Fig. 4**). $^{**}P < 0.01$; one-way ANOVA (**a**, **b**, compared with the corresponding as-printed structures); mean ± s.d. (n = 40).

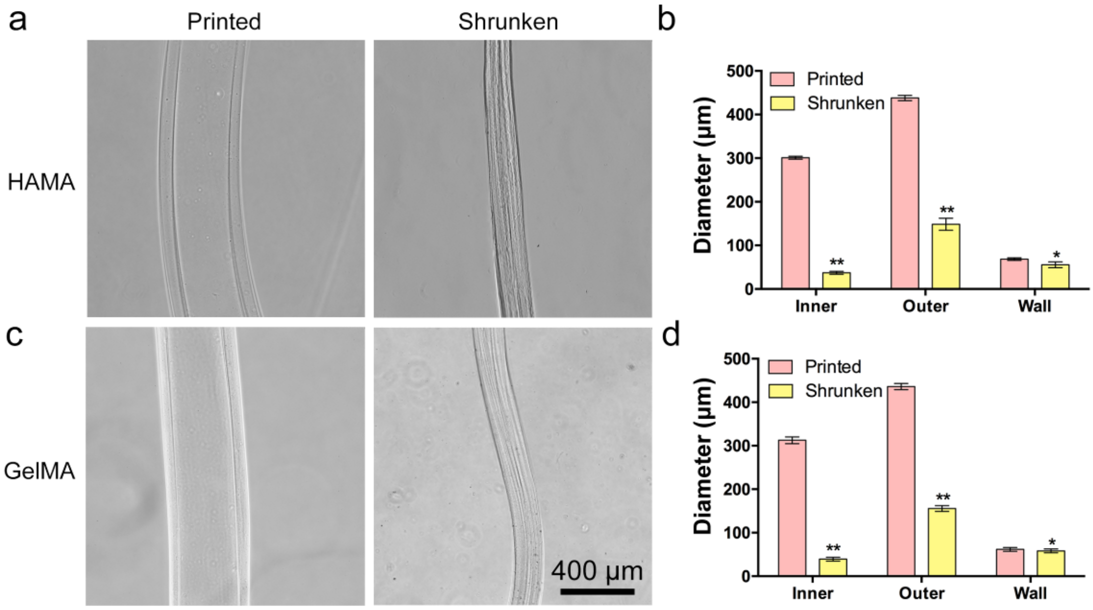


**Supplementary Figure 27.**

**Coaxial printing of small size cannular constructs and their shrinking behavior. a**, Micrographs showing the coaxial printing of cannular HAMA-based constructs before (left) and after (right) 24 h of shrinkage in 2.0 w/v% $HM_w$ chitosan dissolved in 1.0 v/v% acetic acid aqueous solution. **b**, Corresponding quantitative analyses of diameter (inner diameter, outer diameter, wall thickness) changes before and after shrinkage of HAMA-based constructs. **c**, Micrographs showing the coaxial printing of cannular GelMA-based constructs before (left) and after (right) 24 h of shrinkage in 2.0 w/v% $HM_w$ chitosan dissolved in 1.0 v/v% acetic acid aqueous solution. **d**, Corresponding quantitative analyses of diameter (inner diameter, outer diameter, wall thickness) changes before and after shrinkage of GelMA-based constructs. **b**, **d**, $*P < 0.05$, $**P < 0.01$; two-tailed Student's *t*-test (**b**, **d**, compared with the corresponding as-printed structures); mean ± s.d. (n = 40).

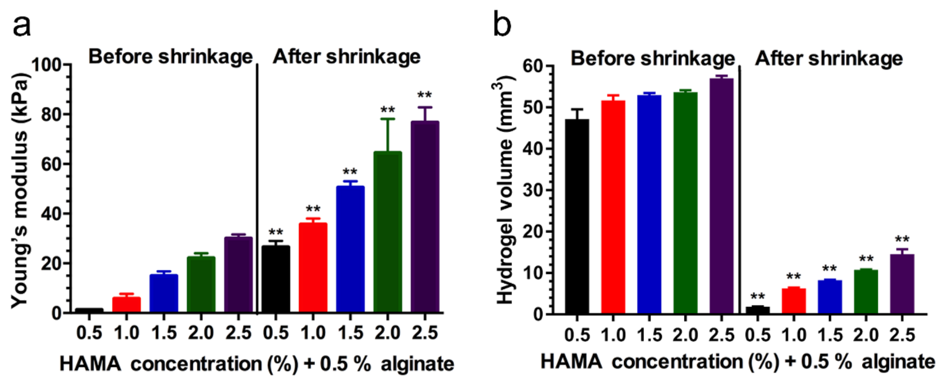


**Supplementary Figure 28.**

**Changes in Young's modulus and volume of HAMA/alginate hydrogel constructs. a**, Change in Young's modulus of HAMA hydrogels containing 0.5 w/v% alginate made with different concentrations of HAMA before and after shrinking in 2.0 w/v% $HM_w$ chitosan. **b**, Change in volume of HAMA hydrogels containing 0.5 w/v% alginate made with different concentrations of HAMA before and after shrinking in 2.0 w/v% $HM_w$ chitosan. **$P < 0.01$; one-way ANOVA (compared with the values of corresponding as-prepared samples); mean ± s.d. (n = 3 or 4).

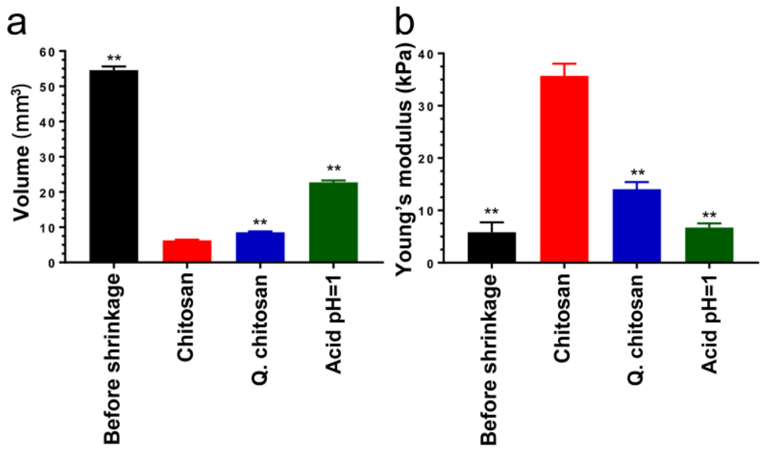


**Supplementary Figure 29.**

**Changes in volume and Young's modulus of HAMA/alginate hydrogel constructs. a**, Change in volume of 1.0 w/v% HAMA/0.5 w/v% alginate hydrogels before and after shrinkage in $HM_w$ chitosan, Q. chitosan (both dissolved at 2.0 w/v % in 1.0 v/v % acetic acid aqueous solution), and an aqueous solution of perchloric acid at pH 1.0. **b**, Change in Young's modulus of 2.0 w/v% HAMA/0.5 w/v% alginate hydrogels before and after shrinkage in $HM_w$ chitosan, Q. chitosan (both dissolved at 2.0 w/v % in 1.0 v/v % acetic acid aqueous solution), and an aqueous solution of perchloric acid at pH 1.0. $^{**}P < 0.01$; one-way ANOVA (compared with the group shrunken in the chitosan solution); mean ± s.d. (n = 3 or 4).

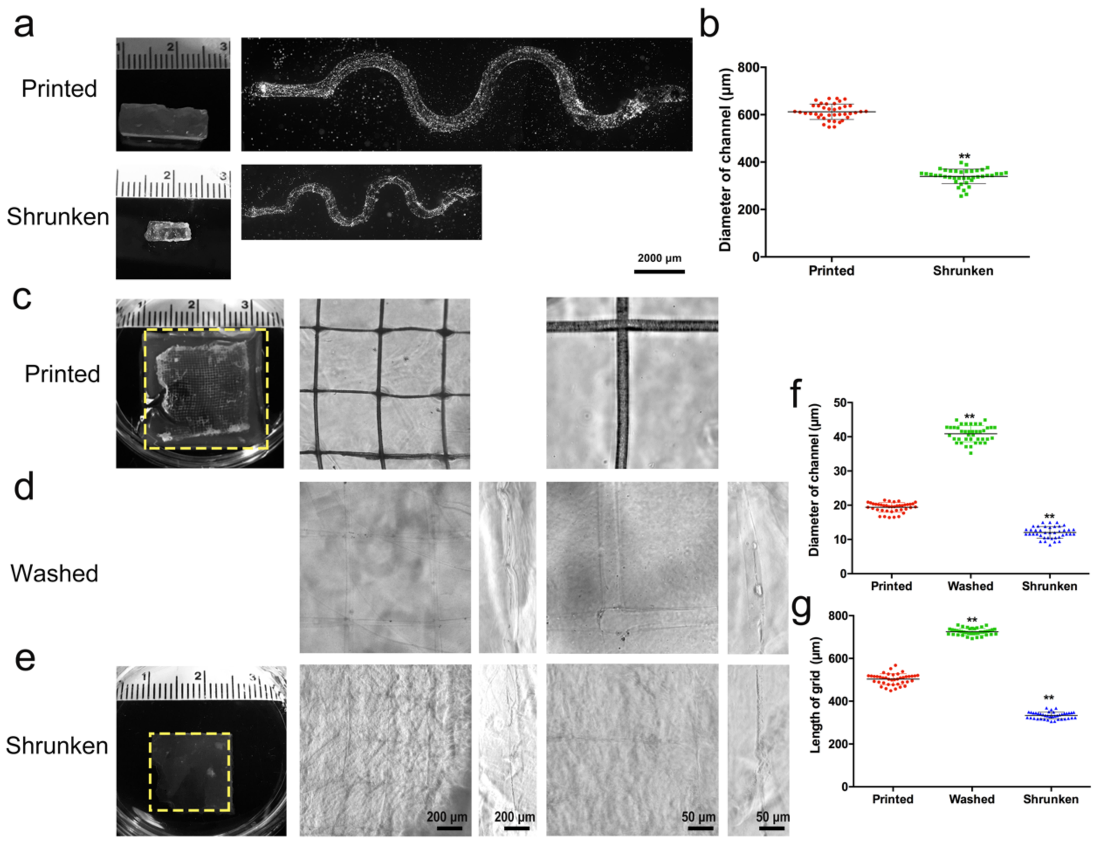


**Supplementary Figure 30.**

**Sacrificial printing of microchannel-embedded GelMA constructs and their shrinking behavior. a**, Photographs showing the size change of the GelMA construct (5.0 w/v%) along with the embedded microchannel before (upper) and after (lower) 24 h of shrinkage in 2.0 w/v% $HM_w$ chitosan dissolved in 1.0 v/v% acetic acid aqueous solution. **b**, Corresponding quantitative analysis of diameter change of the microchannel before and after shrinking. **c**, **d**, **e**, Photographs (left) and micrographs (right) showing the size change of the GelMA construct (5.0 w/v%) along with the embedded microchannel printed (**c**), washed (**d**) and shrunken (**e**) in 2.0 w/v% $HM_w$ chitosan dissolved in 1.0 v/v% acetic acid aqueous solution (24 h). **f**, **g**, Corresponding quantitative analyses of changes in (**f**) diameter of the microchannel and (**g**) length of the grid before and after shrinking. $^{**}P < 0.01$; two-tailed Student's *t*-test (**b**), one-way ANOVA (**f**, **g**, compared with the corresponding as-printed structures); mean ± s.d. (n = 40).

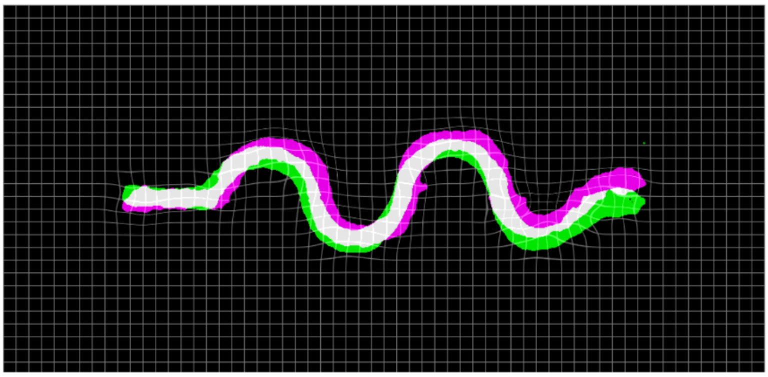

**Supplementary Figure 31.**

Vector-field map of the sacrificially printed microchannel-embedded GelMA hydrogel (relating to **Supplementary Fig. 30a**), comparing the post-shrinking image (magenta) to the corresponding pre-shrinking image (green) by a B-spline-based non-rigid registration algorithm, where the overlaps appear in white and the grids show local distortions. Note that the pre-shrinking images have been rescaled to match the sizes of the post-shrinking images to enable comparisons.

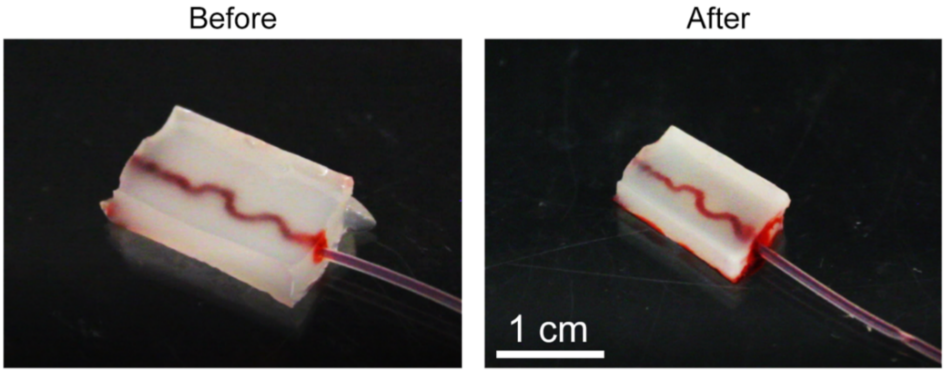


**Supplementary Figure 32.**

Photographs showing perfusion of the same embed sacrificially printed microchannel-embedded GelMA/HAMA hydrogel construct (**a**) before and (**b**) after shrinking in 2.0 w/v% $HM_w$ chitosan dissolved in 1.0 v/v% acetic acid aqueous solution. After perfusion before shrinking, the construct was immersed in a large volume of water to wash out all the dyes to facilitate subsequent shrinking and re-perfusion.

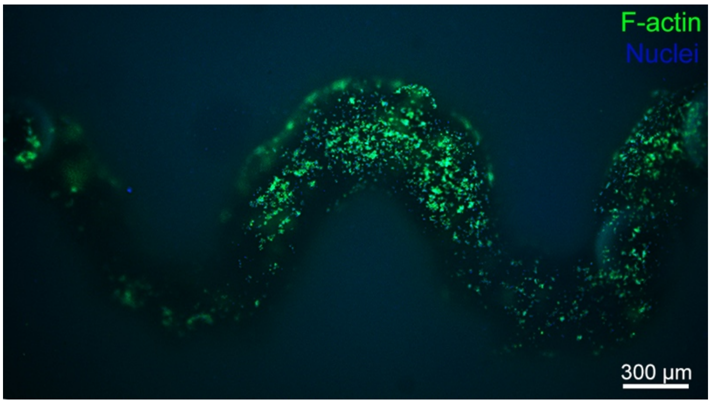


**Supplementary Figure 33.**

Presence of HUVECs on the internal surfaces of a shrunken microchannel in an embedded sacrificially printed GelMA/HAMA hydrogel construct, at day 5, stained for F-actin (green) and nuclei (blue).

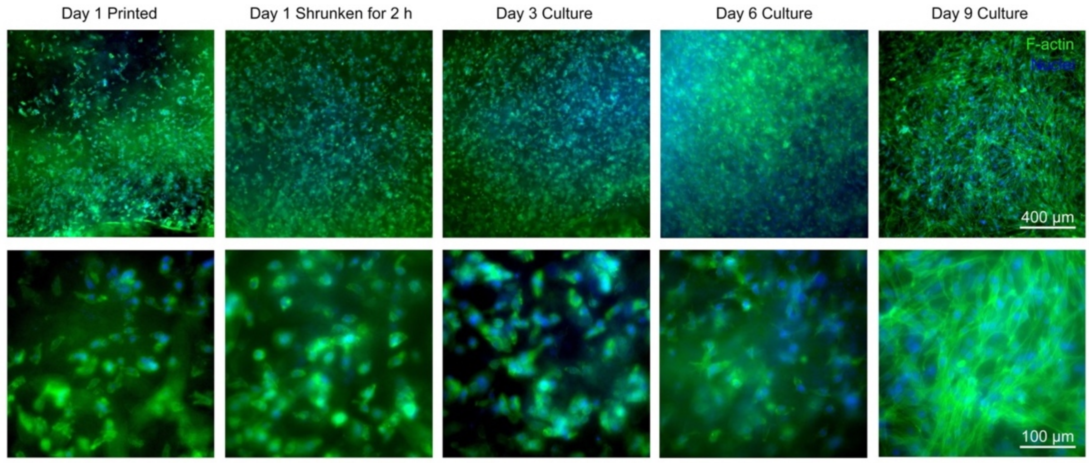


**Supplementary Figure 34.**

The spreading of C2C12 embedded in the GelMA/HAMA constructs before and after shrinking. Morphological observation of C2C12 embedded in mixed hydrogel before and after shrinkage for 1, 3, 6, and 9 days, stained for F-actin (green) and nuclei (blue).

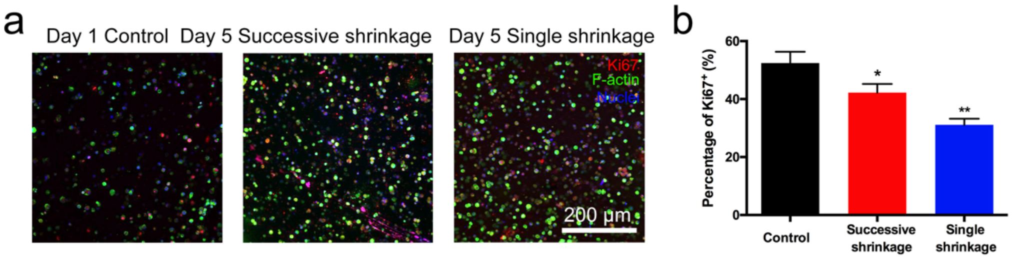


**Supplementary Figure 35.**

**a**, Fluorescence micrographs of HUVECs in GelMA/HAMA constructs without any treatment (control) as well as after successive shrinkage and single shrinkage, stained for Ki67 (red), F-actin (green), and nuclei (blue). **b**, Corresponding quantitative analyses of the percentages of $Ki67^{+}$ stained nuclei in the three groups. *$P < 0.05$, **$P < 0.01$; one-way ANOVA (compared with the control group on Day 1); mean ± s.d. (n = 1, deviations obtained from 8 distinct layers of a confocal stack for each sample).

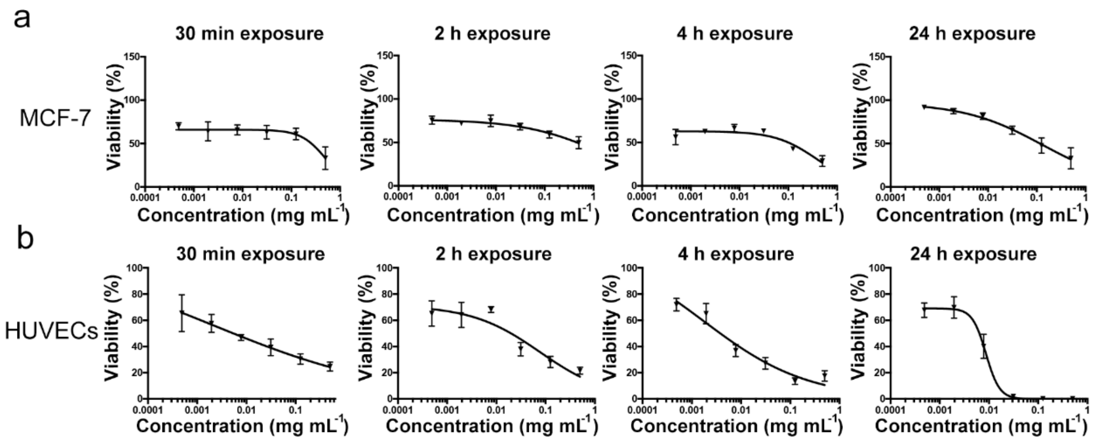


c

| TC$_{50}$ values in Q. chitosan solutions (mg mL$^{-1}$) | | | | |
|---|---|---|---|---|
| Exposure time | 30 min | 2 h | 4 h | 24 h |
| MCF-7 | 0.505 | 1.487 | 0.371 | 0.131 |
| HUVECs | 0.002 | 0.077 | 0.002 | 0.009 |

**Supplementary Figure 36.**

**a,** The viability of MCF-7 cells exposed to different concentrations of Q. chitosan solutions in culture medium for 30 min, 2 h, 4 h, and 24 h. **b,** The viability of HUVECs exposed to different concentrations of Q. chitosan solutions in culture medium for 30 min, 2 h, 4 h, and 24 h. **c,** The calculated TC$_{50}$ values of Q. chitosan for MCF-7 cells and HUVECs. Mean ± s.d. (n = 2 for MCF-7, n = 4 for HUVECs).

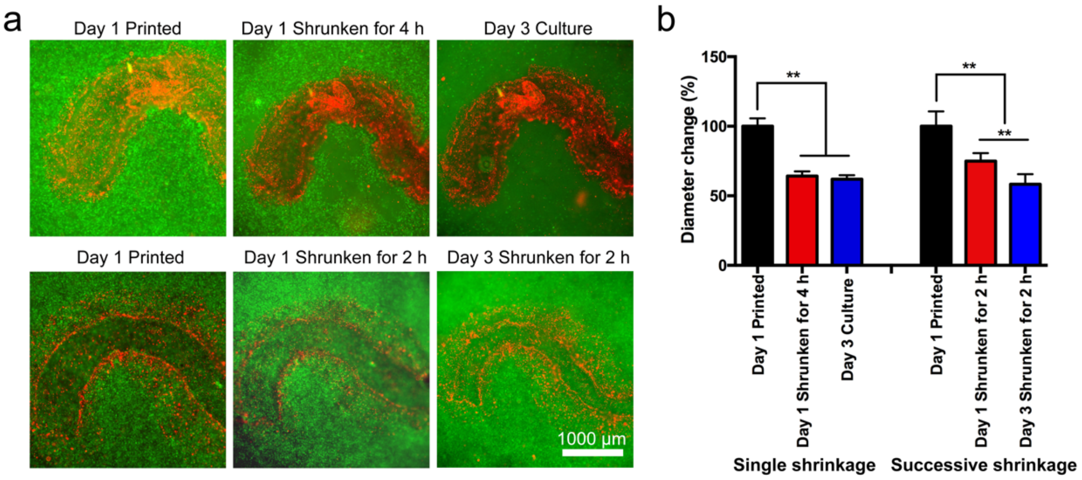


**Supplementary Figure 37.**

**a**, Micrographs showing the changes of the microchannel diameters (red) and the GFP-HUVECs densities (green) in sacrificially bioprinted GelMA/HAMA constructs using Pluronic F-127 as the fugitive bioink, following single shrinkage (upper) and successive shrinkage (lower). Pluronic F-127 was stained in red prior to removal to aid visualization of the microchannels. **b**, Corresponding quantitative analyses of microchannel diameter changes. $^{**}P < 0.01$; one-way ANOVA; mean ± s.d. (n = 10).

**Supplementary Table 1**.

Characteristics of the different types of chitosan and the shrinkage ratios of the HAMA hydrogels.

| Shrinking agent | Deacetylation degree[1] (%) | Quaternization degree[1] (%) | Polymer $M_w$[1] (kDa) | Theoretical average charge per monomer | Average positive charge density[2] (+1 per *x* Da) | Hydrogel[3] volume after shrinking (%) |
|---|---|---|---|---|---|---|
| Chitosan | 85 | 0 | ~15 | 0.62 | 271.8 | 28.9 ± 1.1 |
| | 85 | | 50-190 | | | 21.6 ± 0.9 |
| | 85 | | 700-800 | | | 11.0 ± 0.7 |
| | 72.5 | | 100-250 | 0.53 | 328.8 | 23.6 ± 1.0 |
| | 77.8 | | | 0.56 | 298.7 | 18.5 ± 0.8 |
| | 94.6 | | | 0.69 | 238.3 | 12.6 ± 0.7 |
| Q. chitosan | 90 | 95 | 50-100 | 1.6 | 163.4 | 9.7 ± 0.4 |

[1] per suppliers’ information

[2] at pH 4.7 (1.0 v/v% acetic acid aqueous solution), with a pKa of 6.5 for chitosan amine.

[3] HAMA hydrogels (1.0 w/v%) incubated in 2.0 w/v% of the chitosan dissolved in 1.0 v/v% acetic acid aqueous solution for 24 h.
