# Supplementary material for: Complexation-induced resolution enhancement of 3D-printed hydrogel constructs
Source: Nat Commun. 2020 Mar 9;11:1267. doi: 10.1038/s41467-020-14997-4 (PMC7062888; doi:10.1038/s41467-020-14997-4)
Supplement: Supplementary file 2 — Description of Additional Supplementary Files [file 41467_2020_14997_MOESM2_ESM.pdf]

Description of Additional Supplementary Files

File Name: Supplementary Movie 1
Description: The shrinking process of a printed 2.0 w/v% HAMA hexagon, immersed in 2.0 w/v% HMw chitosan dissolved in 1.0 v/v% acetic acid aqueous solution. The top HAMA hexagon served as the control. The movie is replayed at a speed of 198X

File Name: Supplementary Movie 2
Description: The shrinking process of coaxial-printed tubes with inks containing (upper) 1.0 w/v% HAMA and 0.5 w/v% alginate and (lower) 5.0 w/v% GelMA and 0.5 w/v% alginate, in 2.0 w/v% HMw chitosan dissolved in 1.0 v/v% acetic acid aqueous solution. The movie is replayed at a speed of 162X

File Name: Supplementary Movie 3
Description: The perfusion of the microchannel in an embed sacrificially printed GelMA/HAMA hydrogel construct prior to shrinking.

File Name: Supplementary Movie 4
Description: The perfusion of the microchannel in an embed sacrificially printed GelMA/HAMA hydrogel construct post-shrinking.”
